# Naringenin as a dual-target modulator of RAGE–NF-κB signaling and Aβ fibrillization in Alzheimer’s disease: integrated computational and experimental analyses

**DOI:** 10.3389/fphar.2026.1901236

**Published:** 2026-08-25

**Authors:** Inderjeet Bhogal, Ivo Frydrych, Ajay Modi, Vaishali Pankaj, Akhil Kumar, Amit Khairnar, Viswanath Das, Sudeep Roy

**Affiliations:** 1 Department of Biomedical Engineering, Faculty of Electrical Engineering and Communication, Brno University of Technology, Brno, Czechia; 2 Institute of Molecular and Translational Medicine, Faculty of Medicine and Dentistry, Palacký University and University Hospital Olomouc, Olomouc, Czechia; 3 International Clinical Research Centre, St. Anne University Hospital, Brno, Czechia; 4 Amity School of Biological Sciences, Amity University Punjab, Mohali, Punjab, India; 5 International Clinical Research Centre, Faculty of Medicine, Masaryk University, Brno, Czechia; 6 Department of Physiology, Faculty of Medicine, Masaryk University, Brno, Czechia; 7 Institute of Molecular and Translational Medicine, Czech Advanced Technologies and Research Institute, Palacký University Olomouc, Olomouc, Czechia

**Keywords:** Alzheimer’s disease, Aβ aggregation, dual-target inhibitors, naringenin, neuroinflammation, RAGE signaling

## Abstract

**Introduction:**

Alzheimer’s disease (AD) is a complex neurodegenerative disorder characterized by the accumulation and aggregation of β-amyloid (Aβ) and chronic neuroinflammation mediated by the receptor for advanced glycation end products (RAGE). Current therapeutic candidates targeting RAGE signaling or Aβ aggregation continue to face challenges, including off-target toxicity, poor blood-brain barrier (BBB) penetration, and limited multi-target efficacy.

**Methods:**

Naringenin (NAR), a naturally occurring flavanone polyphenol prioritized through structure-based virtual screening and preliminary biological evaluation, was selected for further mechanistic investigation as a potential dual-target modulator of RAGE-associated inflammatory signaling and Aβ42 fibrillization.

**Results:**

Molecular docking analyses showed that NAR interacts with the RAGE interface (PDB: 6XQ3) via π-cation interactions with ARG_98 and LYS_110 (docking score: −5.072 kcal/mol). At the Aβ fibril (PDB: 2MXU), NAR showed a superior docking score than ALZ-801 (valiltramiprosate), involving hydrogen bonding and π–π stacking with HIS_A14 (docking score: −8.098 kcal/mol). 100 ns molecular dynamics simulations supported the stability of these binding modes, with RMSD and RMSF values within acceptable ranges. MM-GBSA rescoring indicated competitive predicted binding free energies (ΔGBind: −30.260 kcal/mol at RAGE; −220.162 kcal/mol at the fibril, compared to ALZ- 801’s −214.482 kcal/mol), influenced by electrostatic and hydrophobic interactions. Per-residue energy decomposition indicated PHE_20 (−9.10 kcal/ mol) as the dominant hotspot residue, alongside ARG_98 (−4.71 kcal/mol) and LYS_52 (−3.96 kcal/mol) as key RAGE anchors. *In silico* ADMET profiling suggested favorable predicted BBB accessibility (score: 0.001), Caco-2 permeability, Lipinski compliance, and a more favorable predicted cardiac safety profile (hERG: 0.145) relative to V6Y. Experimentally, NAR reduced Aβ42-induced NF-κB activation in RAGE-positive C6 cells, protected SH-SY5Y neurons from Aβ42 toxicity, and inhibited Aβ42 fibrillization *in vitro*, producing non-seeding aggregates consistent with impaired fibril formation, and attenuated rotenone-induced α-synuclein-associated cellular stress.

**Discussion:**

Collectively, these findings support further investigation of NAR as a mechanistically plausible multi-target candidate for protein aggregation and RAGE-associated inflammatory signaling in neurodegenerative disease models.

## 1 Introduction

Alzheimer’s disease (AD) is a major global health issue, primarily due to its increasing prevalence and the profound socioeconomic challenges it presents to modern healthcare systems. This rise is largely attributed to a complex molecular cascade involving the accumulation of extracellular amyloid-β (Aβ) plaques, which trigger neuroinflammation, and the intracellular aggregation of hyperphosphorylated tau protein, which forms neurofibrillary tangles (Dong et al., 2022). Currently, over 55 million people worldwide are living with dementia, and this number is expected to nearly triple by 2050 (Alzheimer’s Association, 2024). As the most common type of dementia, AD represents a significant portion of the global societal and economic burden, costing the economy more than US$ 1.3 trillion annually (Wimo et al., 2017). Beyond the financial impact, AD is a leading cause of disability and caregiver stress due to the continuous progression of symptoms, which involve a decline in memory, thinking, behavior, and social skills. Current symptomatic therapies for AD, including acetylcholinesterase inhibitors and glutamate modulators, provide only temporary symptomatic relief without substantially modifying the underlying disease course. This therapeutic stagnation underscores the urgent need for innovative multi-targeted approaches to address the heterogeneous and interconnected pathology of the disease (Cummings et al., 2025).

Recent studies have identified the receptor for advanced glycation end products (RAGE) as a significant therapeutic biomarker and a key driver of neuroinflammation in AD (Xie et al., 2013). Structurally, RAGE is a 45 kDa transmembrane protein belonging to the immunoglobulin superfamily, characterized by an extracellular region comprising one “V” (variable) domain and two “C” (constant) domains, a single transmembrane helix, and a short cytoplasmic tail (Singh and Agrawal, 2022). RAGE is crucial for transporting Aβ across the blood-brain barrier (BBB) and facilitating inflammatory signaling, which contributes to the neurodegenerative cycle (Chellappa et al., 2020). This pattern-recognition receptor activates the nuclear factor of kappa light-chain enhancer of B-cells (NF-κB) signaling pathway upon binding to ligands such as Aβ and S100 proteins (Deane et al., 2003). This interaction leads to increased oxidative stress and mitochondrial dysfunction, further damaging neuronal integrity and compromising the structural stability of the BBB (Cai et al., 2016). RAGE’s important role in the feedback loop of Aβ accumulation and inflammation has been extensively examined. Its activation inhibits mechanisms involved in Aβ clearance, underscoring its significance in AD pathology (Basta, 2008). Additionally, RAGE is implicated in the limited effectiveness of single-target therapies; simply blocking amyloid does not address the chronic inflammation driven by this receptor (Paudel et al., 2020). These findings underscore RAGE’s multifaceted role in AD biology, making it, along with the Aβ fibril itself, a potential target for new, dual-targeted therapeutic strategies aimed at disrupting both the protein aggregation and signaling-associated inflammation (Burstein et al., 2018). In this context, natural bioactive compounds, particularly flavonoids, offer a promising alternative to synthetic drugs, which are frequently hindered by dose-dependent toxicity.

Natural bioactive compounds, particularly flavonoids, offer a promising and versatile platform for the discovery of potential inhibitors against the complex pathology of AD (Zamanian et al., 2025). These natural substances exhibit pleiotropic properties and can modulate multiple interconnected neuropathological pathways, thereby potentially overcoming some of the limitations associated with synthetic drugs (Calderaro et al., 2022). One notable example is naringenin (NAR), a naturally occurring flavanone with emerging neuroprotective and anti-amyloidogenic potential. NAR has attracted interest due to its favourable physicochemical properties, including reported BBB permeability and a favourable safety profile supporting its investigation for neuroprotective applications (Lai et al., 2025). Preclinical *in vitro* and *in vivo* studies have demonstrated that NAR can reduce oxidative stress and suppress neuroinflammatory signaling pathways (Haider et al., 2020). Additionally, recent *in silico* studies suggest that NAR may interfere with β-sheet-rich fibrillar assemblies, including α-synuclein aggregates in Parkinson’s disease models, supporting its broader relevance to amyloidogenic protein aggregation (Saadh et al., 2024).

The primary goal of this study is to investigate NAR, identified through structure-based virtual screening and preliminary biological evaluation, as a potential dual-target candidate capable of modulating Aβ aggregation and RAGE-mediated inflammatory signaling in AD. To accomplish this, we employed an integrated *in silico* workflow combining structure-based virtual screening, molecular docking, molecular dynamics (MD) simulations, molecular mechanics/generalized born surface area (MM-GBSA) free energy analysis, and absorption, distribution, metabolism, excretion, and toxicity (ADMET) prediction to evaluate the interaction of NAR with both the RAGE receptor and the Aβ fibril relative to reference compounds, including the experimental RAGE inhibitor V6Y and ALZ-801 (valiltramiprosate), a clinically investigated anti-amyloid agent currently in Phase III clinical trials. This dual-target strategy addresses AD as an interconnected pathological network involving both protein misfolding and chronic neuroinflammation rather than as isolated molecular events. Neurodegenerative dementia involves other protein aggregates too, such as α-synuclein (aSyn), one of the main components of lewy body. Therefore, we also explored whether related neuroprotective effects extend to aSyn-associated cellular stress models. Finally, these *in silico* predictions were experimentally evaluated using complementary biophysical aggregation assays and RAGE-associated cellular models to determine whether predicted target engagement correlated with functional suppression of Aβ aggregation and inflammatory neurotoxicity. By integrating computational, biophysical, and cellular approaches, this study aims to evaluate NAR as a mechanistically plausible dual-target candidate that modulates interconnected protein aggregation and neuroinflammatory pathways relevant to AD and related neurodegenerative diseases.

## 2 Materials and methods

### 2.1 In silico

#### 2.1.1 Structure-based virtual screening

To identify novel lead candidates, we implemented an *in silico* virtual screening strategy using the Glide module (Yang et al., 2021) within the Schrödinger Suite, adhering to a hierarchical “funnel” approach to narrow the chemical space. We retrieved a total of 570,725 3D chemical structures in SDF format from a diverse range of repositories, including Enamine’s carboxylic acid fragment library, BACE library, Interbioscreen’s natural and synthetic compounds, and ChemDiv’s drug repurposing library, as detailed in Supplementary Table S1. Structure-based virtual screening of these libraries was conducted using the Virtual Screening Workflow (VSW) of Glide against the RAGE crystal structure (PDB ID: 6XQ3). Before docking, we pre-filtered the prepared libraries based on Lipinski’s rule of five and QikProp properties to ensure an optimal pharmacokinetic profile. Initial structure-based virtual screening using the Glide Standard Precision (SP) method yielded 38,342 hits. Subsequently, all virtual hits from the SP stage underwent more rigorous extra precision (XP) docking-based screening, resulting in a total of 4,270 XP hits. Additionally, we applied a cutoff of −5.0 kcal/mol to filter for high-affinity candidates, resulting in a refined subset of 1,559 molecules with superior Glide GScore. From this subset, the top 20 candidates were post-processed and rescored using the MM-GBSA method. To bridge the gap between computational predictions and biological efficacy, we selected the top six hits based on GScore, MM-GBSA, and QikProp scores for experimental validation.

#### 2.1.2 Protein structure retrieval and preparation

The 3D crystal structures of proteins with PDB IDs: 6XQ3 (human RAGE VC1 domain with V6Y) (Kozlyuk et al., 2021) and 2MXU (42-residue Aβ fibril) (Xiao et al., 2015) were obtained in.pdb format from the Protein Data Bank at https://www.rcsb.org/. They were prepared using Schrodinger Suite 2025-1’s Protein Preparation Workflow (Sastry et al., 2013), which added missing side chains, assigned bond orders, added hydrogen atoms, and created disulfide bonds (Schrödinger, 2025b). Ionization states at pH 7.4 ± 2.0 were generated, and hydrogen bonding was optimized using PROPKA. Structural minimization was done with the OPLS4 force field, removing waters more than 5 Å from heteroatoms.

#### 2.1.3 Ligand structure retrieval and preparation

The ligand structures of NAR (CID: 439246), V6Y (CID: 155937458), and ALZ-801 (CID: 25008296) were retrieved in.sdf format from the NCBI PubChem database (Kim et al., 2016). They were prepared using LigPrep in Schrodinger’s Maestro 14.3 (Schrödinger, 2025a) with the OPLS4 force field at pH 7 ± 2, generating tautomers and determining chiralities, resulting in 32 ligands for each compound.

#### 2.1.4 SiteMap analysis

The potential binding sites of the Aβ fibril were predicted using SiteMap (Halgren, 2009) from Schrödinger’s Maestro 14.3, as it lacked a reference ligand. The process scanned the protein topology with a standard grid, applying a strict hydrophobicity definition and identifying at least 15 site points. We characterized the resulting pockets based on metrics like volume and enclosure, then ranked them using SiteScore. For the RAGE protein, the ligand V6Y bound to the binding site, prompting further study by generating a grid of the binding pocket that occupied V6Y.

#### 2.1.5 Molecular docking

Molecular docking was conducted using the Induced Fit Docking panel (Sherman et al., 2006) of the Prime module to examine the binding of NAR with human RAGE and Aβ fibril. A grid box was created around the co-crystallized ligand (V6Y) in RAGE, and the docking protocol was validated by re-docking this ligand. The NAR molecule was docked into the binding pocket identified by SiteMap for the Aβ fibril. The Standard Precision (SP) approach produced 20 poses for each ligand, followed by the trimming and refinement of side chains based on the B-factor and surrounding backbone residues within 4 Å. After docking, standard molecules, along with NAR, were analyzed for their docking scores and interaction patterns. Additionally, 3D representations of the complexes were generated using the Molecular Operating Environment (MOE) version 2024.0601 (Molecular Operating Environment, 2024).

#### 2.1.6 MD simulations

We conducted MD simulations using Desmond in Schrodinger’s Maestro (Release 2025-1) to evaluate protein-ligand complex stability (Bowers et al., 2006). Each complex was solvated in a 10 × 10 × 10 Å orthorhombic box with SPC water and neutralized with 0.15 M Na+ and Cl- ions. We performed 100 ps of energy minimization, followed by 100 ns of equilibration under an NPT ensemble at 300 K and 1.01325 bar, utilizing the OPLS4 force field. Trajectories were analyzed for RMSD, RMSF, and protein-ligand contacts using the Simulation Interaction Diagram.

#### 2.1.7 Free energy calculations and per-residue decomposition analysis using MM-GBSA

MM-GBSA free energy calculations were performed using Schrodinger’s Prime module. MD simulation trajectories were used to compute binding free energy and total energy of the complex via the thermal_mmgbsa.py script (Genheden and Ryde, 2015). Per-residue energy decomposition analysis identified critical ligand-binding residues by quantifying contributions of individual amino acids (Thapa and Raghavachari, 2019). This was achieved using the breakdown_MMGBSA_by_residue.py script, which calculates average free energy contributions for each residue.

#### 2.1.8 ADMET analysis

ADMETlab 3.0 (Fu et al., 2024), based on a Directed Message Passing Neural Network coupled with the RDKit 2D descriptor, assesses ADMET characteristics, including pharmacokinetics and drug-likeness. This online platform predicts 119 ADMET properties, including physicochemical, pharmacological, and ADME-Tox properties.

### 2.2 In vitro

#### 2.2.1 Materials

Ultra-pure recombinant human Aβ1–42 (Aβ42) peptide in HFIP formulation (Cat. No. AG968, Sigma-Aldrich, St. Louis, MO, United States) was used in all expertoxicity, includingilized peptide was dissolved in anhydrous DMSO to prepare a 5 mM stock solution, aliquoted, and stored at −80 °C until use. Aliquots were thawed on ice and centrifuged at 21,000 × g, 4 °C, 5 min to remove any fibrillar aggregates before use.

ALZ-801 (valiltramiprosate) was obtained from MedChemExpress (Cat # HY-117259; Monmouth Junction, NJ, United States). Naringenin (NAR) was obtained from ENAMINE LV SIA (Cat. ID: EN300-303163, Riga, LV-1073, Latvia) and reconstituted as a 10 mM stock in anhydrous DMSO. Stocks were diluted into phosphate-buffered saline (PBS, pH 7.4) immediately before use, maintaining a constant final DMSO concentration of ≤1% (v/v) across all reactions.

ThT (Cat. No. T3516, Sigma-Aldrich) was prepared as a 1 mM stock in PBS and stored at −20 °C, protected from light. All buffers were prepared with ultrapure Milli-Q water and filtered through 0.22 µm membranes.

#### 2.2.2 ThT aggregation kinetics assay

Aggregation kinetics of Aβ42 (10 µM) were monitored using a ThT fluorescence assay in a clear-bottom, black 384-well ViewPlate (Revvity, Waltham, MA, United States; Part # 6007460) sealed with a TopSealA-PLUS (Revvity) to prevent evaporation, as described previously (Goud et al., 2025). Reactions contained 10 μM Aβ42, 20 μM ThT, and 1% DMSO in PBS (pH 7.4). NAR (1–100 µM) or ALZ-801 (1–100 µM) was added from 10 mM DMSO stocks to maintain a constant solvent concentration.

For spontaneous aggregation, Aβ42 monomer was incubated without pre-formed seeds. For seeded reactions, 0.10 µM pre-formed Aβ42 fibril seeds (monomer-equivalent; 1% of total Aβ) were added from drug-free stock prepared in parallel aggregation reactions (see below). Plates were sealed, incubated at 37 °C, and shaken orbitally at 500 rpm in an EnVision plate reader (PerkinElmer). ThT fluorescence was recorded at Ex 440 nm/Em 485 nm every 3–5 min.

#### 2.2.3 Preparation of pre-formed Aβ fibrils and dot blot analysis

Monomeric Aβ42 (10 µM) was aggregated in PBS (pH 7.4) containing 20 µM ThT at 37 °C with orbital shaking (500 rpm) until the ThT fluorescence plateau was reached. Aggregated samples were pooled and centrifuged at 14,000 × g for 30 min at 4 °C to pellet fibrils, and the supernatants were collected for control analysis. Pellets were resuspended in an equal volume of cold PBS and briefly sonicated (10 s) to redisperse aggregates, yielding 10 µM monomer-equivalent fibril stocks.

When drug-treated samples were prepared, a single wash with cold PBS was performed to remove unbound compound while preserving drug-associated fibrils. Pellets were centrifuged again, resuspended to the original volume, and briefly sonicated (10 s). These insoluble fractions were used for dot blotting and to prepare seed stocks for subsequent assays.

For dot blotting, equal volumes of the resuspended insoluble fractions were spotted in two steps onto nitrocellulose membranes (Bio-Rad; Cat. #1620147) pre-marked with uniform circular grids. Membranes were air-dried (10 min), blocked for 1 h in 5% (w/v) BSA in TBS with 0.05% Tween-20 (TBST), and incubated overnight at 4 °C with β-Amyloid (D54D2) XP® Rabbit mAb (Cell Signaling Technology; Cat. #8243T) diluted 1:1,000 in 5% BSA/TBST. After three washes in TBST, membranes were incubated for 1 h at room temperature in the dark with Alexa Fluor™ 647 Goat anti-Rabbit IgG (Thermo Fisher Scientific; Cat. #A-21245) diluted 1:2000 in 5% BSA/TBST.

Blots were imaged on a ChemiDoc MP imaging system (Bio-Rad) and quantified by integrated density analysis in ImageJ (RRID: SCR_003070). Each dot represented one independent biological replicate.

#### 2.2.4 *In vitro* seeding assay

To evaluate the seeding competence of fibrils formed in the presence or absence of inhibitors, pre-formed fibril seeds were obtained as described above from control, 100 µM NAR-treated, or 100 µM ALZ-801-treated Aβ42 aggregation reactions. Four wells per condition were pooled, centrifuged, washed once in cold PBS to remove unbound drug, re-pelleted, and resuspended to the original volume. The suspension was briefly sonicated (10 s) and used as a 10 µM monomer-equivalent seed stock.

Aggregation reactions contained 10 µM monomeric Aβ42, 20 μM ThT, and 0.10 µM pre-formed fibril seeds (1% of total Aβ). Components were added sequentially in the order PBS, ThT, seeds, then Aβ monomer. Plates were sealed and incubated at 37 °C with orbital shaking (500 rpm). “Seed-only” controls (without monomer) were included to determine background fluorescence. ThT fluorescence was recorded at Ex 440 nm/Em 485 nm every 3 min using an EnVision plate reader (PerkinElmer). Baseline ThT signal (∼2 RFU) reflected background from ThT and seed material and did not affect kinetic comparisons.

#### 2.2.5 C6 glioma cell culture, NF-κB reporter assay, and RAGE expression

C6 glioma cells were maintained in DMEM supplemented with 10% fetal bovine serum (FBS), 1% penicillin–streptomycin, and 1% L-glutamine at 37 °C in a humidified 5% CO_2_ incubator. For RAGE expression analysis, C6 and Chinese hamster ovary (CHO) cells were lysed in (Thermo Scientific; Cat# 89,901) supplemented with protease inhibitors (Cat. #04693116001, Roche), and protein concentrations were determined using a Pierce™ BCA Protein Assay Kit (Thermo Fisher Scientific Inc., Cat. #23,225). Equal amounts of protein were resolved by 10%–12% SDS-PAGE and transferred to PVDF membranes using the Trans-Blot Turbo Transfer System (Bio-Rad). Membranes were blocked in 5% non-fat dry milk in Tris-buffered saline with 0.1% Tween® 20 detergent (TBST), incubated with rabbit anti-RAGE polyclonal antibody (OriGene, Cat# TA327041; 1:5,000), selected based on specificity optimization against multiple commercial anti-RAGE antibodies, and mouse anti-β-actin (Cat. # A2228, Sigma-Aldrich; 1:4,000), followed by anti-rabbit or anti-mouse HRP-conjugated secondary antibodies (Sigma-Aldrich; 1:10,000). CHO lysates served as a RAGE-negative control to verify antibody specificity. Bands were visualized by chemiluminescence on a ChemiDoc MP imaging system (Bio-Rad).

An NF-κB reporter cell line (C6-NF-κB) was generated by lentiviral transduction using the Cignal NF-κB Pathway Reporter construct (Qiagen; Cat. No. 336841), which encodes firefly luciferase downstream of NF-κB–responsive elements. Single-cell clones were isolated by FACS (Aria II, BD Biosciences), functionally validated for NF-κB inducibility, and the clone exhibiting the strongest dynamic response was selected for all experiments. For inhibitor testing, C6-NF-κB cells were seeded into 384-well white opaque plates (PerkinElmer) at a density of 8,000 cells per well and allowed to adhere overnight. Test compounds (NAR 0.1–10 μM; BMS-345541 at 10 µM) were dispensed from DMSO stock solutions using an Echo acoustic liquid handler (Labcyte) to achieve a final DMSO concentration of 0.1%. After a 30-min pre-incubation with compounds, cells were stimulated with Aβ peptide (3.5 µM final concentration). BMS-345541 was included as a positive inhibitory control in parallel wells. Following 6 h of stimulation, BriteLite reagent (PerkinElmer) was added as per the manufacturer’s instructions, and luminescence was measured on an EnSpire Multimode Plate Reader (PerkinElmer). NF-κB activity was expressed as relative light units normalized to Aβ-stimulated controls.

#### 2.2.6 Immunofluorescence staining and quantification of NF-κB p65 localization

C6 cells were seeded on poly-D-lysine–coated 96-well glass-bottom plates (CellVis, Cat. # NC0536760) and allowed to attach overnight. Cells were pre-treated with NAR or vehicle for 30 min, followed by stimulation with Aβ peptide for 6 h. After treatment, cells were fixed in 4% paraformaldehyde and blocked in 5% bovine serum albumin/PBS containing 0.25% Triton X-100 for 1 h at room temperature. Cells were incubated overnight at 4 °C with NFkB p65 Monoclonal Antibody (2A12A7) (1:200 dilution; Invitrogen; Catalog # 33-9900; LOT # ZE392307) diluted in 1% BSA/PBS with 0.05% Triton-X-100, washed three times with PBS, and then incubated for 1 h at room temperature in the dark with anti-Mouse IgG (H + L) Highly Cross-Adsorbed Alexa Fluor™ 488 Secondary Antibody (1:500 dilution; Thermo Fisher Scientific, Cat# A-21202, Lot # 2563848). Nuclei were stained with Hoechst-33342 (Invitrogen, Cat. #H21492), and fluorescence images were captured on a Cell Voyager CV7000 high-content imaging platform (Yokogawa Electric Corporation, Musashino-shi, Tokyo, Japan) using either 20× or ×40 objectives. p65 immunofluorescence was recorded with the 488-nm excitation channel (λexc: 460–490 nm, λem: 500–550 nm), and nuclear staining was detected with the 405-nm excitation channel (λexc: 360–400 nm, λem: 410–480 nm). Images were processed in Signal Image Artist (Revvity, Waltham, MA, United States) using an analysis script that segmented nuclear and cytoplasmic regions of interest and extracted mean fluorescence intensities for each compartment. Nuclear-to-cytoplasmic (N/C) ratios of p65 fluorescence were then calculated on a per-cell basis. A minimum of 70 cells was quantified for each condition across independent experiments.

#### 2.2.7 SH-SY5Y cell culture, Aβ42 oligomer preparation, and viability assay

SH-SY5Y neuroblastoma cells were grown in DMEM/F12 supplemented with 10% FBS and 1% penicillin–streptomycin at 37 °C in a humidified 5% CO_2_ incubator, as described previously (Devyatov et al., 2025). Aβ42 oligomers were generated from the HFIP-treated Aβ42 peptides by dilution into ice-cold serum-free DMEM/F12 to 100 μM, followed by incubation at 4 °C for 24 h without agitation, following established cold-incubation oligomerization protocols (Lambert et al., 1998; Dahlgren et al., 2002). After incubation, the oligomer preparation was centrifuged at low speed to remove visible particulates, and the clarified supernatant was diluted in complete medium to a final concentration of 2.5 μM Aβ42 oligomers for cell treatment. The diluted solution was used on the same day.

For viability experiments, into white OptiPlate 96-well plates (Part # 6002290, Revvity) at a density of 1.2 × 10^4^ cells per well and allowed to attach overnight. The following day, cells were treated with NAR at concentrations of 25 μM, 6.25 μM, 1.56 μM, 0.39 μM, and 0.10 μM in the presence of 2.5 μM Aβ42 oligomers. Control wells included 0.25% DMSO-only control, Aβ42 oligomers alone, and 1 µM staurosporine as a positive cytotoxicity control. After a 24-h incubation period, cell viability was quantified using the RealTime-Glo MT Cell Viability Assay (Promega) according to the manufacturer’s protocol. The luminescence was measured using an Infinite 200 PRO multimode plate reader (Tecan Trading AG, Switzerland) with an integration time of 500 ms. Viability values were normalized to those of DMSO-treated control cells, and protection from Aβ42 oligomer-induced toxicity was calculated relative to the condition with oligomers only.

### 2.3 Statistical and data analysis

All ThT aggregation experiments were independently repeated at least twice using freshly prepared Aβ42 batches. Each condition was run in triplicate for spontaneous and seeding assays and in duplicate for dose–response measurements. Fluorescence traces were analyzed using GraphPad Prism (version 10, San Diego, CA, United States). AUC was calculated from each replicate trace to quantify total fibril formation, as described previously (Goud et al., 2025). Statistical comparisons were performed using one-way ANOVA followed by Dunnett’s or Tukey’s multiple-comparison test with statistical significance accepted at p < 0.05.

## 3 Results

Based on the virtual screening workflow, the top six candidate compounds were further evaluated for cytotoxicity (Supplementary Table S2) and inhibition of Aβ42-induced RAGE–NF-κB signaling in C6 glioma cells (Supplementary Figure S1). Among these candidates, NAR demonstrated the most favourable overall biological profile and was therefore selected for detailed mechanistic investigation.

### 3.1 Protein-ligand interaction analysis: structural basis for the dual-target efficacy of NAR with RAGE and Aβ fibril

Molecular docking was performed for NAR and the respective reference ligands: V6Y (RAGE) and ALZ-801 (Aβ Fibril) against two AD-associated targets: RAGE (PDB: 6XQ3) and Aβ Fibril (PDB: 2MXU). The docking scores and key protein–ligand interactions are summarized in Supplementary Tables S3, S4.

#### 3.1.1 RAGE (6XQ3)—docking results

At the RAGE binding site, the co-crystallized reference ligand V6Y achieved a docking score of −7.033 kcal/mol, establishing the most extensive interaction network among all four complexes. This network includes four hydrogen bonds (with LYS_39, LYS_52, ARG_98, and ASN_112), two salt bridges (involving LYS_39 and LYS_52), and a π-cation interaction with LYS_110. The formation of dual salt bridges with LYS_39 and LYS_52, along with hydrogen bond interactions involving ARG_98 and ASN_112, demonstrates V6Y’s ability to anchor deeply within the positively charged RAGE ligand-binding domain, simultaneously exploiting multiple electrostatic anchoring points (Supplementary Tables S3, S4).

**FIGURE 1:**
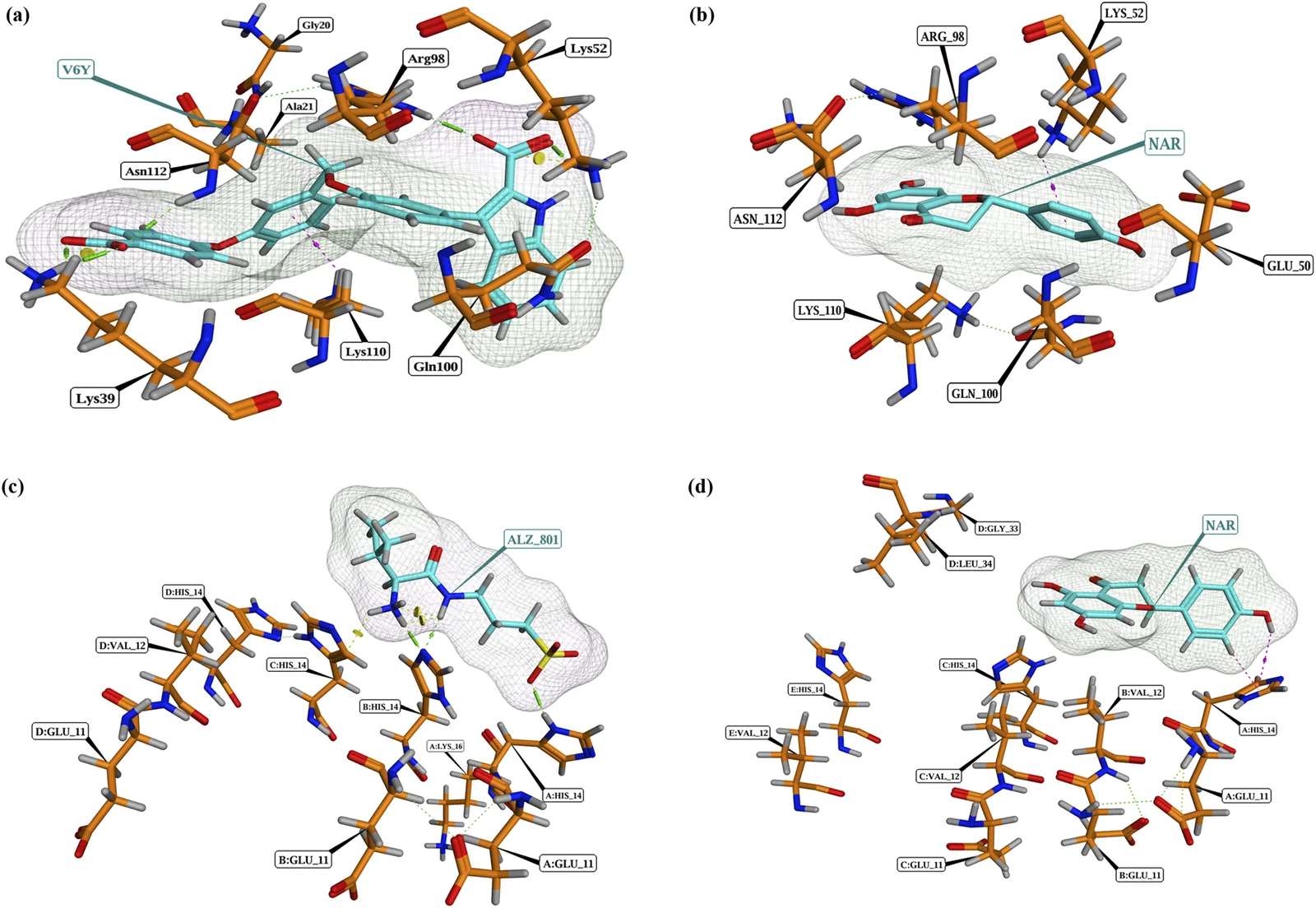
Molecular docking interaction models of RAGE and Aβ42 complexes with reference ligands and NAR. 3D ligand–protein interaction diagrams of RAGE (PDB: 6XQ3) in complex with **(a)** V6Y and **(b)** NAR, and Aβ42 (PDB: 2MXU) in complex with **(c)** ALZ-801 and **(d)** NAR. Protein residues are shown as orange sticks, while ligands are represented as cyan-coloured sticks. Dotted green lines indicate hydrogen-bond interactions with protein backbone residues, dotted pink lines represent H–π interactions, and dotted yellow lines indicate van der Waals (vdW) contacts/clashes contributing to ligand binding orientation.

NAR at RAGE achieved a docking score of −5.072 kcal/mol, which is approximately 2 kcal/mol lower than that of V6Y. Despite this lower score, NAR forms two targeted interactions: a hydrogen bond with GLU_50 and another hydrogen bond with LYS_110, along with π-cation interactions with both ARG_98 and LYS_110. The π-cation interactions result from NAR’s aromatic flavanone ring system engaging with the positively charged side chains of ARG_98 and LYS_110. This type of non-covalent interaction enhances binding affinity and selectivity in aromatic-rich binding pockets. Notably, ARG_98, identified as the dominant hotspot residue in the MM-GBSA decomposition, interacts with NAR via a π-cation interaction with LYS_52 rather than a direct hydrogen bond. This suggests that NAR positions its aromatic scaffold close to ARG_98’s guanidinium group rather than making direct polar contact (Supplementary Table S3), as shown in Figures 1a,b.

#### 3.1.2 Aβ Fibril (2MXU)—docking results

At the Aβ fibril binding site, NAR achieves the highest docking score across all four complexes (−8.098 kcal/mol), surpassing the reference compound ALZ-801 (−6.909 kcal/mol) by approximately 1.2 kcal/mol. This finding corroborates the MM-GBSA results (NAR: −220.162 kcal/mol vs. ALZ-801: −214.482 kcal/mol) and collectively establishes NAR as the superior binder at the Aβ fibril site (Supplementary Table S3).

ALZ-801 forms two hydrogen bonds, one with HIS_A:14 and another with HIS_B:14 from adjacent β-sheet chains and does not engage in any additional non-covalent interactions. This cross-chain hydrogen bond interaction between both A and B chains is pharmacologically significant, indicating that ALZ-801 bridges the fibril protofilament interface and may disrupt fibril stability. However, the lack of hydrophobic or π-system interactions reduces its overall docking score (Figure 1c).

NAR interacts with the Aβ fibril by forming a hydrogen bond with HIS_A:14 and engages in a π-π stacking interaction with the same residue. This dual engagement leverages both the hydrogen-bond donor and acceptor capabilities of the histidine imidazole ring and its aromatic π-system. The combination of hydrogen bonding and aromatic stacking with HIS_A:14 is a distinctive feature of NAR’s planar chromone scaffold, representing a particularly energetically favorable binding mode. The π-π stacking with HIS_A:14 is supported by per-residue decomposition data, which identifies HIS_A:13 as a significant hotspot, influenced by van der Waals (−5.83 kcal/mol) and lipophilic (−1.43 kcal/mol) contributions typical of aromatic stacking geometries (Figure 1d; Supplementary Tables S3–S5).

### 3.2 MD simulations over 100 ns support stable NAR interactions with RAGE and Aβ fibril

To capture the temporal dynamics of ligand binding and validate the stability of the predicted docking poses, 100 ns MD simulations were conducted in 10 replicates for all four protein–ligand complexes: 6XQ3-V6Y, 6XQ3-NAR, 2MXU-ALZ-801, and 2MXU-NAR. However, the results presented are based on 10 independent replicas, which provide a more robust design for assessing reproducibility and consistency than a single 500 ns trajectory. Simulation Interaction Diagrams (SIDs) were created to visualize the persistence nature of ligand contacts within the active site throughout the simulation trajectories, offering dynamic support for the static docking and MM-GBSA binding profiles.

#### 3.2.1 RAGE interaction persistence

**FIGURE 2:**
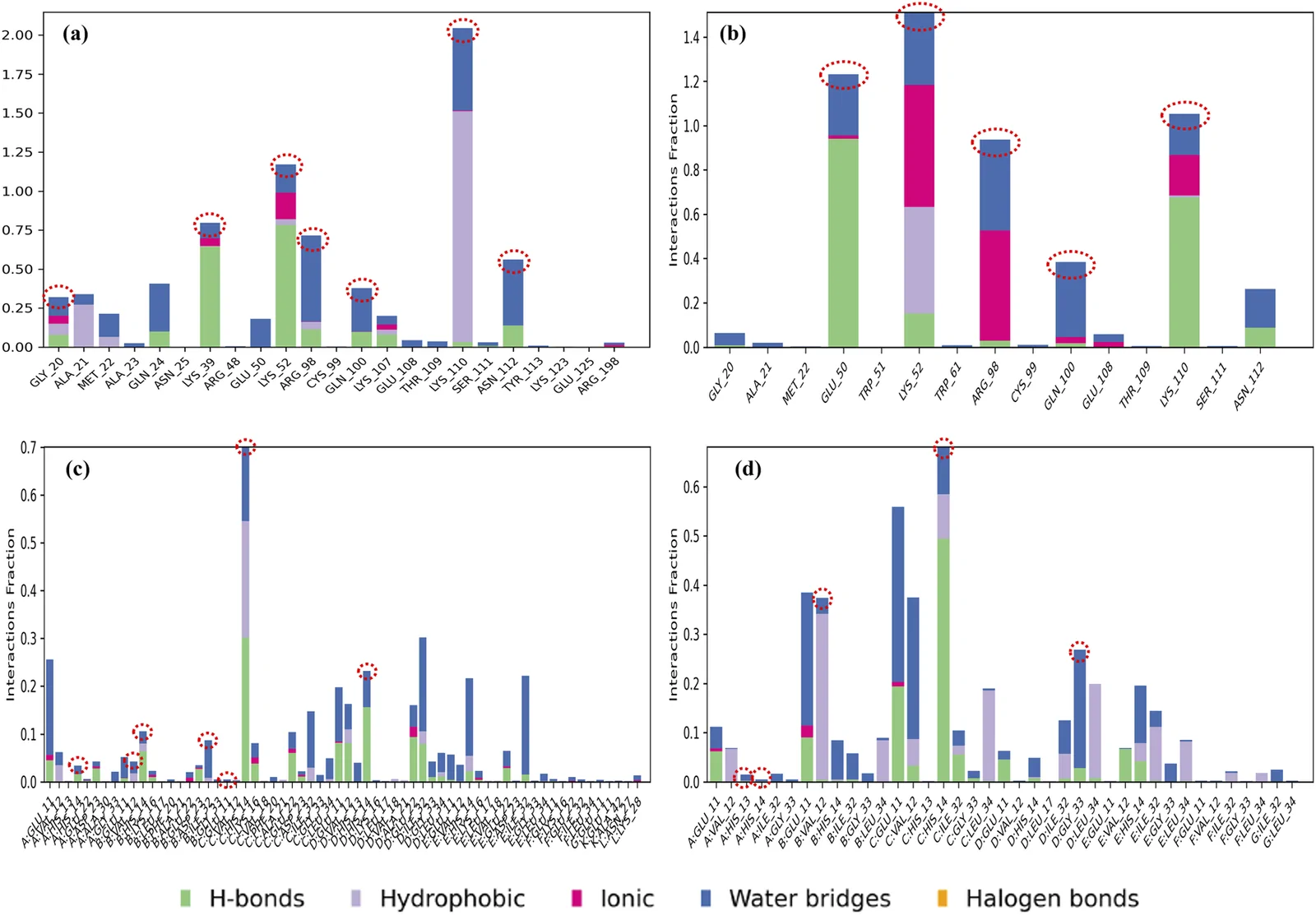
Protein-ligand interaction profiles from 100 ns molecular dynamics simulations of RAGE and Aβ fibril complexes. Protein-ligand contact profiles and interaction histograms illustrating the molecular mechanisms of the **(a)** RAGE (PDB: 6XQ3) in complex with the native ligand (V6Y), and **(b)** RAGE bound to the natural flavanol NAR. Panels **(c)** and **(d)** depict the Aβ fibril (PDB: 2MXU) in complex with the clinical candidate ALZ-801 and NAR, respectively. All trajectories were analysed over a 100-ns MD timescale to assess structural convergence and binding mechanism. The residues encircled in red play critical roles in defining the stability, affinity, and mechanism of action.

In the RAGE complex (6XQ3-V6Y), stability relies on hydrogen bonds with LYS_39 and LYS_52, water bridges at ARG_98, LYS_110, and ASN_112, a key hydrophobic contact with LYS_110, and ionic interactions at GLY_20, LYS_39, and LYS_52 (Figure 2a). The 6XQ3-NAR complex features hydrogen bonds with GLU_50 and LYS_110, strong ionic attractions at LYS_52, ARG_98, and LYS_110, a hydrophobic interaction with LYS_52, and water bridges involving GLU_50, LYS_52, ARG_98, and GLN_100 (Figure 2b).

#### 3.2.2 Aβ fibril interaction persistence

In contrast, the 2MXU-ALZ-801 complex shows weaker, transient interactions, mainly water-mediated contacts between GLU_11 (chain B) and HIS_14 (chain B), with few stable interactions. It forms multiple hydrogen bonds with HIS_14 (chain A), GLU_11 (chains C and D), and VAL_12 (Chains A and D) (Figure 2c; Supplementary Table S5). The 2MXU-NAR complex, however, forms persistent contacts with Aβ, targeting HIS_14 (chain B) through strong hydrogen and hydrophobic bonds, and involves VAL_12 (chain B) and GLY_33 (Chain B) to mediate water interactions, indicating a stabilizing binding mode of NAR within the Aβ fibril (Figure 2d; Supplementary Table S5).

Further analysis indicated that Protein-RMSD values were within the acceptable range of 1–4 Å (Supplementary Figure S2). However, L-RMSD profiles for the 2MXU-ALZ-801 and 2MXU-NAR complexes showed significant changes compared to the 6XQ3-V6Y and 6XQ3-NAR complexes (Supplementary Figure S2). Protein-RMSF calculations revealed all protein-ligand complexes maintained adequate values within 2 Å, indicating stability (Supplementary Figure S3). The simulation interaction diagrams were consistent across all replicate trajectories.

### 3.3 Free energy calculations and per-residue decomposition analysis reveal the energetic basis of dual target binding of NAR

Binding affinities and stability of protein-ligand complexes were assessed using the MM-GBSA method, which decomposes the total binding energy into components: Coulombic, covalent, hydrogen bond, lipophilic, solvation (GB), and van der Waals energies. This analysis examines interactions between V6Y and NAR with RAGE and ALZ-801, and NAR with Aβ, summarized in Table 1. For detailed MM-GBSA analysis from the 100 ns simulation trajectory, including average binding affinities and standard deviations (Supplementary Table S6).

**TABLE 1:** Total binding energy contribution of protein-ligand complexes calculated using MM-GBSA (kcal/mol).

Protein	Ligand	ΔG_Bind_	ΔG_Coulomb_	ΔG_Covalent_	ΔG_Hbond_	ΔG_Lipo_	ΔG_SolvGB_	ΔG_vdW_
RAGE	V6Y	−41.945	−187.300	−1.761	−2.795	−4.303	189.564	−32.395
	NAR	−30.260	−153.963	0.915	−1.609	−5.620	158.034	−27.014
Aβ (fibril)	ALZ-801	−214.482	88.723	1.759	−8.796	−41.139	−99.041	−152.547
	NAR	−220.162	50.351	−0.340	−7.827	−40.051	−77.092	−141.419

Abbreviations: ΔG_Bind_, Total binding free energy averaged over the simulation time; ΔG_Coulomb_, Electrostatic Coulomb term of the binding energy; ΔG_Covalent_, Covalent term of the binding energy; ΔG_Hbond_, Hydrogen bond contribution to the binding energy; ΔG_Lipo_, Lipophilic contribution to the binding energy; ΔG_SolvGB_, Generalized born term of the solvation energy; ΔG_vdW_, van der Waals term of the binding energy.

For RAGE, V6Y exhibited a total binding free energy (ΔGBind) of −41.945 kcal/mol, which was more favorable than NAR (−30.260 kcal/mol), indicating that V6Y binds more strongly to RAGE. The dominant energetic driver for both compounds at RAGE was the Coulomb (electrostatic) interaction; V6Y contributed −187.300 kcal/mol, and NAR −153.963 kcal/mol, both substantially offset by the large desolvation penalty (ΔGSolvGB: +189.564 and +158.034 kcal/mol, respectively). The vdW contributions (−32.395 for V6Y; −27.014 for NAR) and lipophilic interactions (−4.303 for V6Y; −5.620 for NAR) provided secondary stabilizing contributions.

For the Aβ fibril, NAR demonstrated a marginally superior total binding free energy of −220.162 kcal/mol compared to the reference compound ALZ-801 (−214.482 kcal/mol), suggesting that NAR may bind the fibril with slightly greater affinity. In contrast to RAGE, the binding mechanism at the Aβ Fibril was dominated by vdW interactions (NAR: −141.419; ALZ-801: −152.547 kcal/mol) and lipophilic contacts (NAR: −40.051; ALZ-801: −41.139 kcal/mol), reflecting the hydrophobic nature of the amyloid fibril groove. The solvation term (ΔGSolvGB) was favorable for both Aβ complexes (−99.041 for ALZ-801; −77.092 for NAR), contrasting sharply with the large unfavorable desolvation seen at RAGE, and indicative of solvent exclusion upon fibril groove binding.

Furthermore, Per-residue decomposition analysis identified key hotspot amino acids that contribute to ligand binding. The complete energy breakdown for each residue, heteroatoms, and the ligand (UNK) during the 100 ns simulation is in Supplementary Table S5. Energy contributions from the top ten residues are in Supplementary Table S7.

#### 3.3.1 RAGE binding-electrostatic dominance and hotspot comparison

The per-residue decomposition at the RAGE (6XQ3) binding interface reveals that the binding mechanism is overwhelmingly governed by Coulombic electrostatic interactions, particularly at cationic residues LYS_39, LYS_52, ARG_98, and LYS_110. These residues carry positive charges that form strong salt bridges and ion-dipole interactions with the anionic pharmacophore elements in both V6Y and NAR. A critical feature is the large solvation penalty that counteracts each Coulombic contribution; for example, ARG_98 with V6Y shows Coulomb = −22.29 kcal/mol offset by Solvation = +19.42 kcal/mol, reflecting the energetic cost of desolvating these charged residues upon ligand binding. The net dGbind of −7.35 kcal/mol for ARG_98 confirms it as the dominant hotspot for V6Y binding (Supplementary Table S7).

ARG_98 is the most important anchor residue for both V6Y (−7.35 kcal/mol) and NAR (−4.71 kcal/mol) at RAGE, underscoring its critical pharmacological role. NAR engages ARG_98 with an even stronger Coulomb contribution (−25.56 kcal/mol) than V6Y (−22.29 kcal/mol), yet its net binding at this residue is weaker (−4.71 vs. −7.35 kcal/mol) due to proportionally higher solvation penalty, indicating a difference in burial geometry. The residues GLY_20, ALA_21, MET_22, GLN_100, and ASN_112 contribute through vdW and lipophilic contacts, forming a hydrophobic sub-pocket adjacent to the charged interface. NAR engages GLU_50, TRP_51, and TRP_61 in this region through aromatic stacking and vdW interactions, interactions absent in V6Y, highlighting NAR’s unique binding profile at RAGE (Supplementary Table S7).

#### 3.3.2 Aβ fibril binding—hydrophobic core dominance

The binding landscape at the Aβ fibril is fundamentally distinct from that of RAGE, characterized by a dense hydrophobic core and contributions from 22 hotspot residues for both ALZ-801 and NAR, the highest hotspot count across all four complexes. The dominant interaction forces are vdW and lipophilic, consistent with NAR and ALZ-801 intercalating into the amyloid fibril’s hydrophobic steric zipper groove. Residues VAL_12, LEU_17, VAL_18, VAL_24, ILE_31, LEU_34, MET_35, VAL_36, VAL_39, VAL_40, and ILE_41 form a highly conserved hydrophobic shell that both compounds engage extensively (Supplementary Table S7).

The most striking difference between ALZ-801 and NAR at the fibril is the emergence of PHE_20 as the top hotspot for NAR (−9.10 kcal/mol), driven by exceptional vdW (−6.28 kcal/mol), lipophilic (−4.69 kcal/mol), and π-π stacking (−0.89 kcal/mol) interactions. This residue is absent from ALZ-801’s hotspot set, indicating NAR extends deeper into the aromatic sub-pocket of the fibril groove, engaging in a π-π stacking interaction with PHE_20 that confers additional stabilization. This may explain NAR’s slightly superior total ΔGBind (−220.162 kcal/mol) compared to ALZ-801 (−214.482 kcal/mol) observed in the MM-GBSA results. Similarly, PHE_19 contributes −1.56 kcal/mol for NAR through vdW (−4.14 kcal/mol), and lipo (−2.97 kcal/mol) contacts another aromatic residue exclusively engaged by NAR.

HIS_13 and HIS_14 are shared hotspots between ALZ-801 and NAR, with NAR engaging HIS_13 more strongly (−4.80 vs. −3.06 kcal/mol), driven by higher vdW (−5.83 vs. −4.43 kcal/mol) and lipophilic (−1.43 vs. −0.97 kcal/mol) contributions; suggesting deeper burial of NAR’s aromatic chromone ring system within the HIS_13-flanked binding sub-pocket. The glutamine residue GLN_15 serves as a critical polar anchor for both compounds, providing a mixed Coulomb + vdW + H-bond contribution (ALZ-801: −6.30; NAR: −5.16 kcal/mol), likely through its side-chain amide engaging with the compound’s hydroxyl groups (Supplementary Table S7).

### 3.4 Comparative *in silico* ADMET profiling reveals distinct pharmacokinetic and safety characteristics among NAR, V6Y, and ALZ-801

#### 3.4.1 Physicochemical properties and drug-likeness

The physicochemical characteristics of all three compounds are summarized in Supplementary Table S8. NAR displays a balanced physicochemical profile with a molecular weight (MW) of 272.07 g/mol (within the optimal 100–600 g/mol range), a TPSA of 86.99 Å^2^, well within the ≤140 Å^2^ threshold, and only one rotatable bond, indicating a relatively rigid and compact scaffold. Its logP of 2.477 falls within the optimal 0–3 range, reflecting moderate lipophilicity compatible with membrane permeation. Its logS of −3.831 indicates moderate aqueous solubility, remaining within the generally acceptable range for oral compounds. V6Y presents a larger MW of 479.14 g/mol and a logP of 5.890, marginally exceeding Lipinski criteria, and receives a Lipinski violation score of 0, suggesting it remains within the border of oral drug-likeness. ALZ-801 has the smallest MW (238.1 g/mol) and a negative logP (−1.627), indicating a comparatively hydrophilic profile and a high predicted aqueous solubility (logS = −0.346). None of the three compounds trigger PAINS filters (score = 0), indicating the absence of obvious reactive or promiscuous structural motifs that would interfere with biological assays.

#### 3.4.2 Absorption

The predicted absorption profile differed across the three compounds. NAR shows the highest predicted Caco-2 permeability (−4.964), followed by V6Y (−5.076), whereas ALZ-801 (−5.936) fell below the −5.15 threshold in the computational. MDCK permeability was low for all three compounds, suggesting limited passive transcellular transport. Both NAR and V6Y showed excellent HIA (0.001), whereas ALZ-801 showed a comparatively higher HIA value (0.386), potentially reflecting differences in physicochemical properties and transport behavior (Supplementary Table S8). Since ALZ-801 has already advanced into clinical evaluation, these predictions should be interpreted primarily as comparative computational descriptors relative to NAR and V6Y rather than standalone indicators of its absorption behavior.

#### 3.4.3 Distribution

The distribution profile is particularly relevant given the CNS-associated targets evaluated in this study. All three compounds demonstrate predicted BBB penetration scores ≤0.003, suggesting CNS accessibility. However, NAR and V6Y exhibit high plasma protein binding (PPB) values of 94.963% and 98.656%, respectively, which may reduce the circulating free drug fraction available for target engagement. ALZ-801 shows a substantially lower predicted PPB (21.706%), consistent with its clinically developed CNS-directed profile and suggesting a comparatively larger free-circulating fraction despite its lower predicted lipophilicity (Supplementary Table S8).

#### 3.4.4 Metabolism

NAR and V6Y were predicted to be non-inhibitors of CYP2D6, suggesting a lower likelihood of CYP2D6-mediated drug–drug interactions. However, NAR shows a relatively high probability of being a CYP2D6 substrate (0.850), suggesting potential hepatic metabolism via this pathway. In contrast, V6Y and ALZ-801 showed minimal predicted CYP2D6 substrate probability (0.005 and 0.000, respectively), suggesting comparatively greater predicted metabolic stability at this isoform (Supplementary Table S8). For ALZ-801, these computational findings are broadly consistent with its existing pharmacokinetic characterization in clinical development.

#### 3.4.5 Excretion

NAR demonstrates the highest predicted clearance rate (7.019) among all three compounds, followed by ALZ-801 (5.009) and V6Y (0.031), suggesting comparatively faster predicted elimination. The extremely low predicted clearance of V6Y (0.031) may indicate a greater likelihood of accumulation upon repeated dosing. All three compounds showed predicted half-life values within the lower range (<0.7), suggesting the possibility of relatively rapid systemic elimination (Supplementary Table S8). However, for ALZ-801, these predictions should be interpreted cautiously, given the availability of experimental and clinical pharmacokinetic data.

#### 3.4.6 Toxicity profile

NAR showed an excellent hERG score (0.145), suggesting a favorable cardiac safety profile compared to V6Y (0.377; medium predicted risk). ALZ-801 showed the lowest predicted hERG liability score (0.013), consistent with a favorable predicted cardiac safety profile. All three compounds show moderate predicted Ames mutagenicity risk (0.422–0.682), indicating that experimental mutagenicity testing would be required for confirmation. NAR and ALZ-801 show relatively high predicted skin sensitization scores (0.708 and 0.982, respectively), although this may be less relevant for orally administered neurological candidates than topical exposure. Predicated carcinogenicity risk was moderate for all three compounds (0.358–0.656), and further experimental validation would be required to define this risk (Supplementary Table S8).

While *in silico* profiling suggests that NAR has a favorable pharmacokinetic and preliminary safety profile, computational affinity and predicted ADMET properties alone do not establish biological activity. Therefore, we next evaluated whether NAR could functionally suppress Aβ-induced RAGE-NF-κB signaling in a cellular context. Given that RAGE activation by extracellular Aβ triggers NF-κB-dependent inflammatory signaling, we used a cell-based validation model to test whether the predicted target engagement translated into measurable pathway inhibition.

### 3.5 NAR attenuates RAGE–NF-κB signaling in C6 glioma cells and mitigates Aβ-induced toxicity in SH-SY5Y cells

**FIGURE 3:**
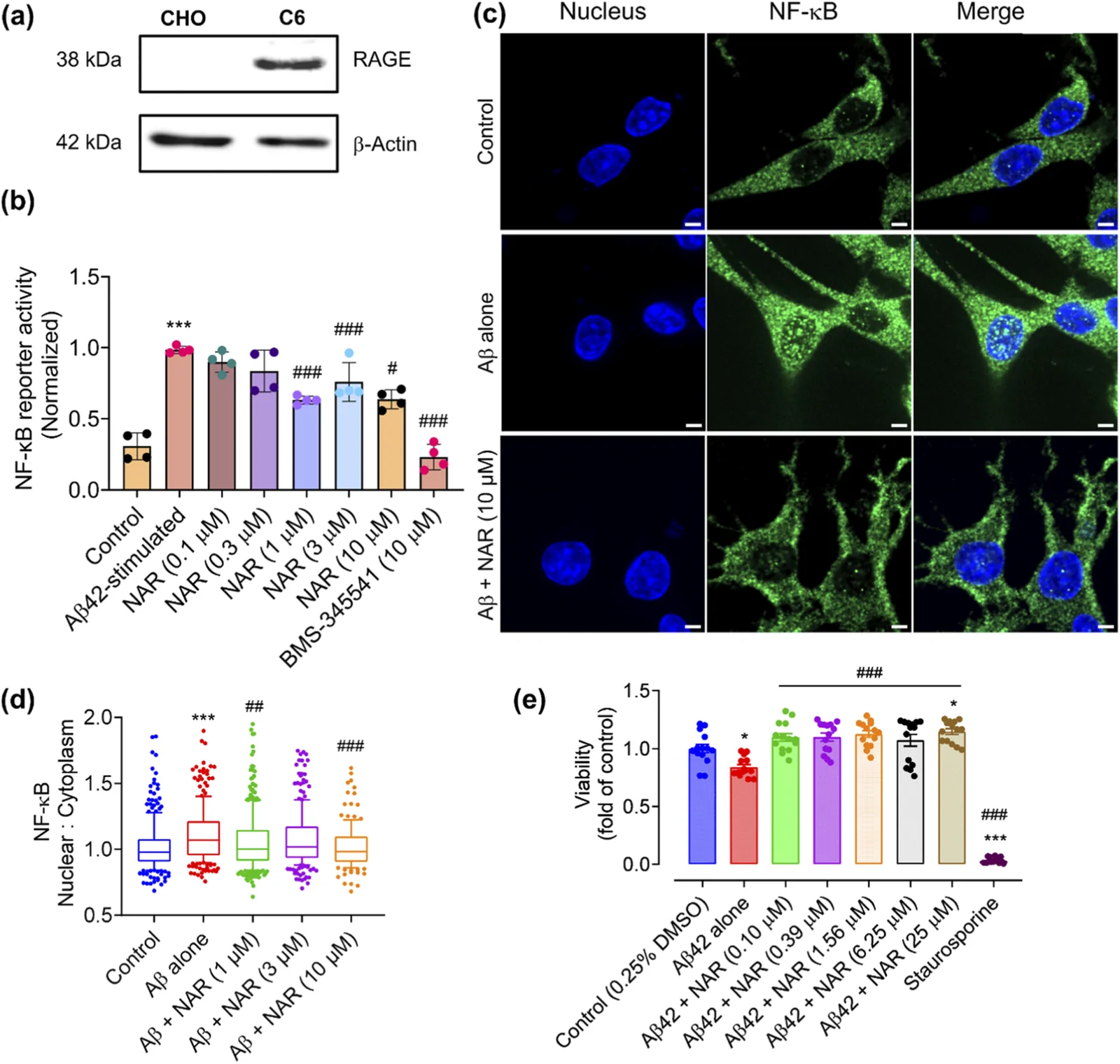
NAR inhibits RAGE-dependent NF-κB signaling and protects against Aβ42-induced toxicity. **(a)** Western blot analysis of RAGE expression in C6 glioma cells, with CHO cells serving as a RAGE-negative control. A clear ∼38 kDa RAGE band is observed in C6 lysates but absent in CHO. β-Actin was used as a loading control. Images of the uncropped blot are shown in Supplementary Material (Supplementary Figure S4). **(b)** NF-κB reporter activity in C6-NF-κB cells pre-treated with NAR (0.1–10 μM, 30 min) followed by stimulation with Aβ42 for 6 h. Luciferase activity was normalized to Aβ42 controls. BMS-345541 served as a positive inhibitory control. Data are mean ± SEM (n = 4). One-way ANOVA with Dunnett’s *post hoc* test; ***p < 0.001 vs. control, ^#^p < 0.05, ^###^p < 0.001 vs. Aβ42-stimulated. **(c)** Confocal microscopy of NF-κB p65 localization shows cytoplasmic staining in unstimulated cells, nuclear accumulation after Aβ42 treatment, and suppression of nuclear translocation with NAR pre-treatment. Scale bar: 20 µm. **(d)** Quantification of NF-κB p65 nuclear translocation showing that NAR prevents Aβ42-induced NF-κB p65 nuclear accumulation. Box-and-whisker plots show 25th–75th percentiles with 5th–95th percentile whiskers; the central line indicates the mean (n > 70 cells per condition from two replicates). ***p < 0.001 vs. control, ^#^p < 0.05, ^###^p < 0.001 vs. Aβ alone. **(e)** Viability of SH-SY5Y cells exposed to Aβ42 oligomers for 24 h with or without NAR. Viability is expressed relative to DMSO-treated controls. Staurosporine (1 µM) was included as a cytotoxicity control. Data are mean ± SEM (n = 3), one-way ANOVA (Tukey’s *post hoc* test), *p < 0.05, ***p < 0.001 vs. control, ^###^p < 0.001 vs. Aβ alone.

To validate the predicted RAGE-targeting activity of NAR, we first confirmed that the C6 glioma cells. Western blot analysis showed a clear ∼38 kDa RAGE band in C6 cells, whereas CHO cells, used as a negative control, lacked detectable RAGE expression, confirming C6 cells as a RAGE-positive system for downstream assays (Figure 3a).

In C6-NF-κB reporter cells, Aβ42 stimulation produced a robust increase in luciferase activity relative to unstimulated controls, indicating strong NF-κB activation. Pre-treatment with NAR reduced this response in a concentration-dependent manner, with inhibition at higher NAR concentrations comparable to the IKK inhibitor BMS-345541 (Figure 3b).

We next examined NF-κB p65 localization by immunofluorescence. Under basal conditions, p65 was predominantly cytoplasmic, whereas Aβ42 stimulation caused clear nuclear accumulation. NAR pre-treatment markedly reduced p65 nuclear translocation and maintained a predominantly cytoplasmic localization pattern (Figures 3c,d).

To assess whether NAR also protects neuronal cells from Aβ42-induced toxicity, SH-SY5Y cells were exposed to Aβ42 oligomers in the presence or absence of NAR. Aβ42treatment significantly reduced cell viability, whereas NAR co-treatment preserved viability at substantially higher levels (Figure 3e).

Additional supplementary experiments were performed in differentiated SH-SY5Y dopaminergic-like cells exposed to rotenone to check whether the neuroprotective effect of NAR extends beyond the AD-related paradigm. Firstly, we checked its cytotoxicity in differentiated SH-SY5Y cells with different concentrations (0–100 μM), and we found that NAR was toxic at 1, 20, 80, and 100 µM concentrations. Thereafter, we checked its efficacy against rotenone, and NAR demonstrated neuroprotection against rotenone at 4 and 10 μM (Supplementary Figure S7). To further explore the molecular effect of NAR, RT-PCR and ICC were performed for the PD-related markers. NAR pretreatment restored the rotenone-decreased tyrosine hydroxylase level (TH; rate-limiting enzyme for the synthesis of dopamine) at both mRNA and protein levels. Moreover, it also reduced the rotenone-induced RAGE expression at both mRNA and protein levels; SNCA mRNA expression and phosphorylation of α-synuclein (an aggregatory protein forming Lewy bodies in PD) (Supplementary Figure S8). All of these findings show that NAR regulates key molecular markers linked to the pathophysiology of PD and protects against rotenone-induced neurotoxicity. These additional findings imply that inhibition of RAGE signalling may offer wider neuroprotection across multiple neurodegenerative disorders, even though AD is the main focus of the current study.

### 3.6 NAR inhibits spontaneous and seeded aggregation of Aβ42 and reduces fibril seeding competence

**FIGURE 4:**
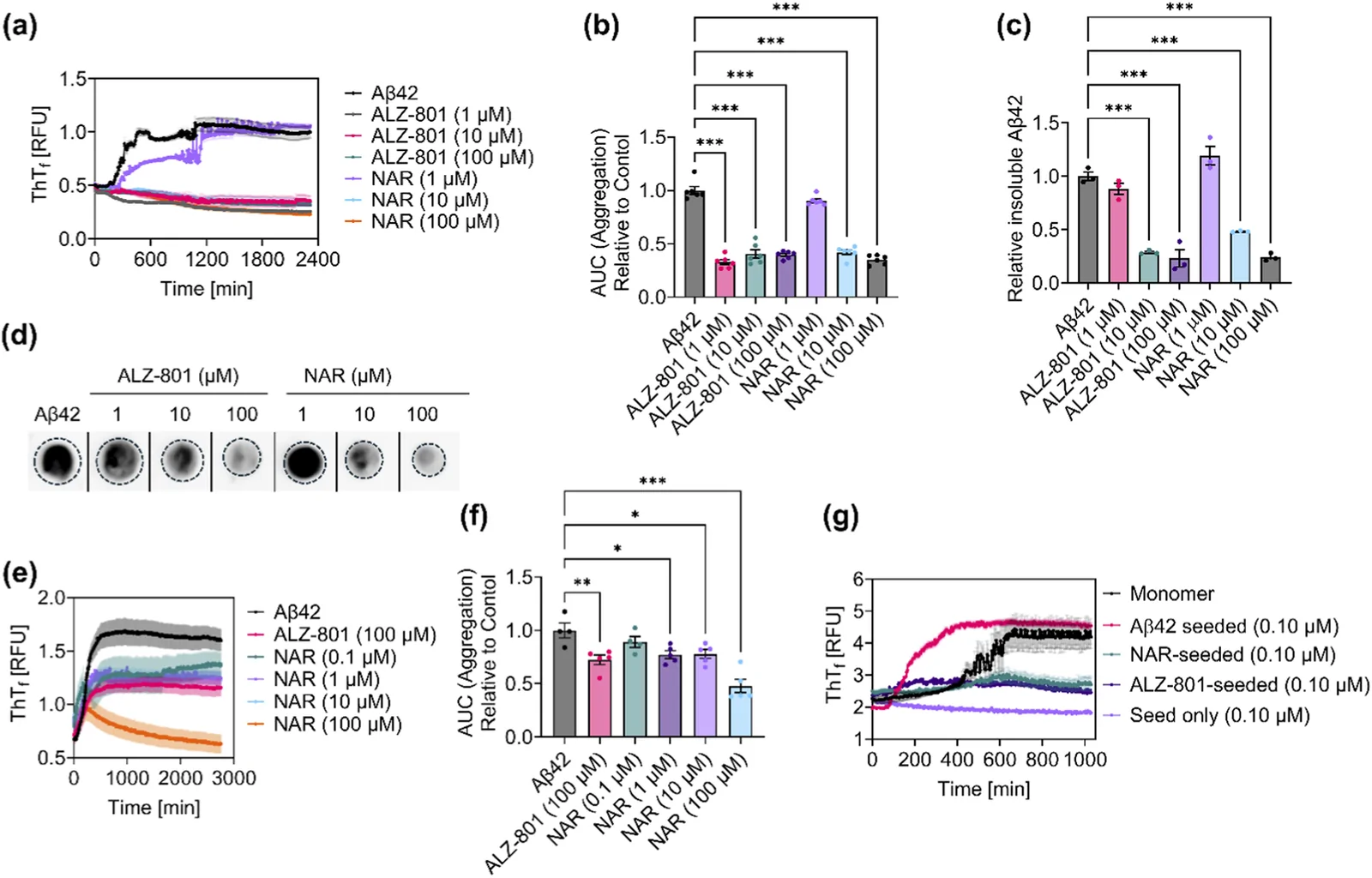
NAR inhibits spontaneous and seeded aggregation of Aβ42, reduces insoluble Aβ accumulation, and prevents secondary seeding. **(a)** ThT fluorescence kinetics of Aβ42 (10 µM) monitored during spontaneous aggregation in the absence or presence of ALZ-801 (1–100 µM) or NAR (1–100 µM). **(b)** Quantitative analysis of spontaneous aggregation expressed as the area under the curve (AUC) from panel **(a)**. Mean ± SEM (n = 3). ***p < 0.001 vs. Aβ42 control, one-way ANOVA with Dunnett’s test. **(c, d)** Quantification and dot-blot analysis of insoluble (pellet) fractions collected at the endpoint of the reactions shown in panel **(a)**. Mean ± SEM (n = 3). ***p < 0.001, one-way ANOVA (Dunnett’s *post hoc* test). Dotted circles indicate regions used for integrated-density quantification. The unmodified dot-blot image is provided in the Supplementary Material (Supplementary Figure S5). **(e)** ThT fluorescence kinetics of seeded aggregation reactions containing 10 µM monomeric Aβ42 and 0.10 µM pre-formed fibril seeds incubated with or without ALZ-801 (100 µM) or NAR (0.1–100 µM). Mean ± SEM (n = 2). **(f)** Quantification of seeded aggregation by AUC analysis of panel **(e)**. Mean ± SEM (n = 2). ***p < 0.001, **p < 0.01, *p < 0.05, one-way ANOVA (Dunnett’s *post hoc* test). **(g)** Seeding competence of fibrils formed in the presence or absence of inhibitors. Monomeric Aβ42 (10 µM) was seeded with 0.10 µM pre-formed fibrils generated in separate reactions without drug (Aβ42), with NAR (100 µM), or with ALZ-801 (100 µM). ThT fluorescence (Ex 440 nm/Em 485 nm) was monitored at 37 °C (500 rpm) in PBS with 20 µM ThT. “Seed-only” controls contained the same seed concentrations without monomer. Mean ± SEM (n = 3). Baseline ThT fluorescence (∼2 RFU) reflects background signal from ThT and seed material and does not affect kinetic comparisons.

Spontaneous aggregation of Aβ42 (10 µM) produced the expected sigmoidal increase in ThT fluorescence, consistent with nucleation-dependent fibrillization (Figure 4a). Both NAR and ALZ-801 inhibited this process in a concentration-dependent manner. ALZ-801 strongly suppressed Aβ42 fibrillization across all tested concentrations, whereas NAR produced marked inhibition at ≥ 10 µM. Area-under-the-curve (AUC) analysis confirmed a significant reduction in total ThT fluorescence for both compounds relative to untreated Aβ42 controls (Figure 4b).

Endpoint dot-blot analysis of insoluble fractions from the same reactions showed reduced Alexa Fluor 647–detected Aβ42 signal in the presence of both inhibitors (Figures 4c,d). Quantification of integrated density demonstrated that ALZ-801 and high-dose NAR substantially reduced pelletable insoluble Aβ42 aggregates.

In seeded aggregation assays containing pre-formed Aβ42 fibril seeds (0.10 µM), control reactions showed rapid fluorescence increase, whereas co-incubation with NAR (0.1–100 µM) or ALZ-801 (100 µM) markedly slowed and reduced ThT signal development (Figure 4e). AUC analysis demonstrated significant, concentration-dependent inhibition by NAR and near-complete suppression by ALZ-801 (Figure 4f), indicating that NAR interferes with both primary fibril formation and seed-driven aggregation.

To determine whether inhibitor-derived fibrils retain seeding competence, aggregates generated in the presence of NAR or ALZ-801 were used as seeds in a fresh Aβ42 aggregation reaction. Control fibrils efficiently accelerated aggregation, whereas fibrils formed in the presence of NAR or ALZ-801 failed to promote rapid fibrillization, indicating impaired seeding competence (Figure 4g).

Together, these findings show that NAR inhibits Aβ42 aggregation, reduces insoluble aggregate accumulation, and disrupts the formation of propagation-competent fibrils.

## 4 Discussion

### 4.1 Molecular docking analysis of NAR interactions with RAGE and Aβ fibril

Molecular docking provided the initial structural insights into NAR’s dual biological activity, suggesting that NAR may modulate both RAGE-associated signaling and amyloid fibril formation. At the RAGE interface (PDB: 6XQ3), NAR achieved a docking score of −5.072 kcal/mol, forming direct hydrogen bonds with GLU_50 and LYS_110 while establishing π-cation interactions with ARG_98 and LYS_110 in the RAGE V-type immunoglobulin domain, which mediates Aβ oligomer recognition. This competitive occupation of the Aβ-recognition interface provides a possible structural explanation for the experimentally observed decrease in Aβ42-induced NF-κB activation in RAGE-positive C6 cells, as NAR targets the same lysine/arginine-rich binding surface that RAGE uses to recognize Aβ42 oligomers. This may reduce RAGE–Aβ42 complex formation and diminish downstream neuroinflammatory signaling. The reference compound V6Y scored higher at −7.033 kcal/mol due to a more extensive polar contact network, incorporating four hydrogen bonds with LYS_39, LYS_52, ARG_98, and ASN_112, in addition to dual salt bridges at LYS_39 and LYS_52, and a π-cation interaction with LYS_110. This reflects V6Y’s design as a specific RAGE modulator; however, its clinical development faced challenges related to toxicity and inadequate CNS penetration, limitations that were considered comparatively during ADMET evaluation of NAR (Figure 1; Supplementary Tables S3, S4).

At the Aβ Fibril (PDB: 2MXU), NAR not only outperformed ALZ-801 in docking score (−8.098 vs. −6.909 kcal/mol) but also engaged the target through a distinct contact mechanism. NAR utilized simultaneous hydrogen bonding and π-π aromatic stacking with HIS_A:14 and GLY_C:33, whereas ALZ-801 relied on bifurcated cross-chain hydrogen bonds with HIS_B:14, which did not involve any π-system engagement (Gazit, 2002). NAR’s π-π stacking with HIS_A:14 may interfere with Cu^2+^/Zn^2+^ coordination sites at positions 13 and 14, which are known to promote amyloid nucleation (Atrián-Blasco et al., 2018). This suggests that NAR may exert inhibitory effects beyond simple groove occupation (Sharma et al., 2013). Overall, docking predictions highlight a shared set of functionally critical residues: GLU_50, LYS_52, ARG_98, GLN_100, and LYS_110 at RAGE (Bhogal et al., 2025), as well as HIS_A:14, GLY_C:33, and hydrophobic groove residues at the fibril. These residues acted as molecular anchors for all subsequent computational layers, which aimed to dynamically confirm and energetically resolve them (Figure 1; Supplementary Tables S3–S5).

### 4.2 MD simulation analysis of NAR binding stability

To determine whether these static binding modes were dynamically maintained under physiologically relevant conditions, 100-ns molecular dynamics simulations were performed in replicates for all four complexes. The protein backbone RMSD values remained stable within the 1–4 Å range throughout (Supplementary Figure S2), and the RMSF values supported retention of overall structures within 2 Å. This suggests that NAR does not substantially perturb the structural integrity of either target, consistent with the lack of significant cytotoxicity observed in SH-SY5Y viability assays at therapeutically relevant concentrations. For the 6XQ3–NAR complex, docking-predicted π-cation interactions with ARG_98 and LYS_110 remained strong across all replicate trajectories, alongside direct hydrogen bonds with GLU_50 and LYS_110, hydrophobic interactions with LYS_52, and water bridges involving GLU_50, LYS_52, ARG_98, and GLN_100 (Figure 2). The consistent presence of ionic interactions at ARG_98 and LYS_52, the structural core of the RAGE Aβ-recognition interface, is consistent with the sustained NF-κB suppression observed in the C6 cellular assay. This suggests that NAR maintains relatively stable occupancy at the RAGE interface over the stimulated timescales (Supplementary Figure S3; Table 1).

At the fibril, the 2MXU–NAR Simulation Interaction Diagram (SID) exhibited the most stable and energetically favorable binding mode across all four complexes. It maintained sustained hydrogen bonding and hydrophobic interactions at HIS_14 (chain A) and GLY_33 (chain C) throughout the 100 ns simulation, along with stable water bridge contacts at VAL_12, LEU_17, and ILE_32, allowing NAR to stay engaged within the fibril groove (Ma et al., 2021). In contrast, the 2MXU–ALZ-801 SID showed primarily transient, water-mediated interactions between GLU_11 (chain A) and HIS_14 (chain C), highlighting ALZ-801’s hydrophilic nature and the lack of π-system contacts (Kocis et al., 2017; Muramatsu et al., 2025). This binding mode aligns with its need for a substantial molar excess to achieve complete inhibition of Aβ42 oligomerization. The consistent presence of NAR’s interactions with the fibril groove across all MD replicates is consistent with the observed reduction in the seeding competence of NAR-treated fibrils (Muramatsu et al., 2025). Continuous aromatic and hydrophobic interactions within the fibril groove may interfere with the inter-strand β-sheet packing geometry required for template-directed elongation (Konstantoulea et al., 2022), resulting in non-fibrillar, ThT-silent, off-pathway Aβ42 assemblies, as confirmed experimentally (Kamalaldinezabadi et al., 2024).

### 4.3 MM-GBSA analysis of binding energetics

MM-GBSA rescoring provided a quantitative thermodynamic framework to explain NAR’s binding selectivity and relative potency across the two targets. At RAGE, the dynamically confirmed electrostatic contacts resulted in binding energetics overwhelmingly dominated by Coulomb interactions for both NAR (−153.963 kcal/mol) and V6Y (−187.300 kcal/mol). These values were significantly offset by large desolvation penalties (+158.034 and +189.564 kcal/mol, respectively). NAR achieved a total ΔGBind of −30.260 kcal/mol, which is 72% of V6Y’s ΔGBind of −41.945 kcal/mol. This energetic relationship is broadly consistent with the NF-κB assay data, in which NAR competitively inhibits inflammatory signaling, albeit less effectively than V6Y. This difference may reflect NAR’s fewer formal salt-bridge contacts and its lower overall Coulomb contribution (Table 1).

In contrast, the energetic landscape at the Aβ fibril was fundamentally different. NAR demonstrated a superior ΔGBind of −220.162 kcal/mol, compared to ALZ-801’s −214.482 kcal/mol, with binding largely influenced by van der Waals forces (−141.419 kcal/mol) and lipophilic interactions (−40.051 kcal/mol). A favorable solvation term of −77.092 kcal/mol was consistent with hydrophobic burial upon intercalation into the fibril groove (Table 1). This thermodynamic signature aligns with the early nucleation stage of Aβ42 aggregation, where hydrophobic collapse contributes to β-sheet formation, and NAR exhibited strong inhibitory effects experimentally.

### 4.4 Per-residue decomposition analysis of hotspot interactions

Per-residue energy decomposition analyzed these binding profiles at a single-residue level, identifying the specific molecular anchors that account for the biological outcomes observed experimentally. At RAGE, ARG_98 stood out as the dominant hotspot for both V6Y (−7.35 kcal/mol; Coulomb: −22.29 kcal/mol) and NAR (−4.71 kcal/mol; Coulomb: −25.56 kcal/mol), representing the most substantial electrostatic contribution at the residue level within the entire dataset. This is consistent with the persistent π-cation interaction between NAR and ARG_98, observed in docking and MD simulations, which contributed the strongest electrostatic single-residue interaction in the system. Additional contributors to NAR’s RAGE hotspot profile include LYS_52 (NAR: −3.96 kcal/mol), LYS_110 (NAR: −1.83 kcal/mol), GLN_100 (NAR: −2.82 kcal/mol), and GLU_50 (NAR: −1.13 kcal/mol) (Figures 3, 4; Supplementary Table S5). Since LYS_52 and ARG_98 are precisely the residues within the RAGE V-domain responsible for recognizing Aβ42 oligomers, NAR’s significant binding contributions at these sites may contribute to the observed reduction in RAGE–Aβ42 complex formation and NF-κB activation in C6 cells.

The decomposition of the Aβ fibril provided an important mechanistic insight into the study. Both NAR and ALZ-801 feature a conserved hydrophobic hotspot network composed of valine, isoleucine, leucine, and methionine residues. However, NAR uniquely interacts with the aromatic phenylalanine sub-pocket through PHE_20 (−9.10 kcal/mol; vdW: −6.28; lipo: −4.69; π-π: −0.89), which represents the highest per-residue contribution across the entire dataset and is absent from ALZ-801’s profile, alongside HIS_13 (−4.80 kcal/mol) and PHE_19 (−1.56 kcal/mol) (Supplementary Figure S6; Supplementary Table S5). PHE_19 and PHE_20 is situated within the hydrophobic core of the Aβ42 β-sheet stacking interface, where established inter-strand F19–F20 aromatic contacts are critical for stabilizing the β-sheet and for competent fibril propagation. NAR’s unique engagement of the π-system at this sub-pocket may interfere with these inter-strand contacts, destabilizing the β-sheet architecture necessary for seeding-competent fibril formation. This interpretation is consistent with the experimentally observed non-seeding aggregate phenotype. This mechanism at the residue level provides a possible molecular explanation for why NAR produces fibrils that lack seeding competence. This finding was initially predicted by docking via π-π stacking, further confirmed by molecular dynamics through persistent hydrophobic contacts, quantified by MM-GBSA as hydrophobic energetic dominance, and specifically attributed to PHE_20 and the aromatic sub-pocket architecture that is inaccessible to ALZ-801 through decomposition analysis.

### 4.5 ADMET profiling and translational considerations

The convergent computational evidence must be evaluated in light of NAR’s pharmacokinetic and toxicological profile, especially considering that the clinical failures of V6Y and the translational limitations reported for ALZ-801 were primarily attributed to ADMET issues rather than purely mechanistic deficiencies. NAR’s physicochemical profile is most compliant with Lipinski’s rule of five, including an optimal molecular weight (272.07 g/mol), logP (2.477), TPSA (86.99 Å^2^), and a single rotatable bond, among other test compounds (Supplementary Table S8). Notably, the molecular rigidity that facilitates NAR’s dual-target binding, allowing simultaneous π-cation interactions at RAGE and aromatic sub-pocket intercalation at the fibril groove, may also contribute to physicochemical properties favorable for oral drug-likeness, suggesting a possible structural relationship between binding behavior and drug-likeness. NAR exhibits predicted BBB penetration, with a score of 0.001, which may help address the CNS permeability limitations associated with V6Y. Additionally, it demonstrates superior Caco-2 permeability (−4.964) compared to ALZ-801 (−5.936, below threshold), coupled with a favorable hERG cardiac safety score (0.145 compared to V6Y’s medium-risk score of 0.377) and the highest clearance rate among all tested compounds (7.019 versus V6Y’s critically low 0.031).

Together, these computational predictions suggest lower predicted risks of cardiac liability and drug accumulation than for V6Y (Supplementary Table S8). The lack of cytotoxicity observed in the SH-SY5Y neuronal viability assay provides preliminary cellular-level support for this favorable tolerability. Two pharmacokinetic liabilities must be considered in the translational pathway: high plasma protein binding (94.963%) reduces the free drug fraction available for CNS target engagement, and a high CYP2D6 substrate probability (0.850) indicates a susceptibility to first-pass hepatic metabolism. These are recognized optimization targets that can be addressed through prodrug development and nanoparticle encapsulation strategies. Their translational impact may be partially mitigated by *in vitro* evidence showing measurable NF-κB suppression in C6 cells and neuronal protection in SH-SY5Y assays. The moderate Ames mutagenicity (0.682) and carcinogenicity (0.656) scores necessitate confirmation through *in vitro* Ames testing, but they are broadly consistent with NAR’s established safety profile as a widely consumed dietary flavanone (Supplementary Table S8).

### 4.6 Experimental validation of NAR activity

The *in vitro* cellular and biophysical findings broadly support the computational observations. NAR reduces Aβ42-induced NF-κB activation in RAGE-positive C6 cells and protects SH-SY5Y neurons from Aβ42 oligomer-mediated toxicity (Figure 3). This is consistent with the predicted interactions involving ARG_98, LYS_52, and LYS_110, which are dynamically sustained across all MD replicates and quantified thermodynamically through Coulomb-dominated MM-GBSA energetics (ΔGBind: −30.260 kcal/mol) with resolution at the residue level through per-residue hotspot contributions. Complementary experiments in differentiated SH-SY5Y dopaminergic-like cells further supported its broader neuroprotective relevance in a rotenone-induced stress model. Modulation targeting RAGE diminished dopaminergic toxicity, restored TH expression, and reduced the level of phosphorylated α-synuclein and RAGE. This suggests that RAGE-associated neuroinflammatory signaling may extend beyond amyloid-driven pathology.

In fibril formation, NAR inhibits Aβ42 fibrillization under both spontaneous and seeded conditions, decreases the accumulation of insoluble aggregates, and produces non-seeding aggregates (Figure 4). This is consistent with docking-predicted intercalation within the fibril groove and persistent hydrophobic contacts during MD simulations and quantifies hydrophobic dominance (vdW: −141.419 kcal/mol; total ΔGBind: −220.162 kcal/mol) through decomposition, identifying engagement in the aromatic sub-pocket at PHE_20, PHE_19, and HIS_13. NAR’s aggregation kinetics align with other flavonoids and polyphenols that redirect Aβ42 towards off-pathway, low-ThT-emitting assemblies by stacking interactions and hydrogen-bonding that disrupt β-sheet interfaces (Granzotto and Zatta, 2011; Al-Edresi et al., 2020; Saadh et al., 2024; Álvarez-Berbel et al., 2025). These interactions are now specifically and energetically mapped to PHE_19, PHE_20, and HIS_13 (Supplementary Table S5) and align with the vdW/lipo-dominated hotspot network involving VAL_12, LEU_17, PHE_19, PHE_20, VAL_24, MET_35, and ILE_41 (Xue et al., 2017).

The convergence of computational, cellular, and biophysical findings supports NAR as a mechanistically plausible dual-target candidate whose observed biological effects are broadly consistent with the predicted interaction framework.

## 5 Conclusion

The integration of molecular docking, MD simulations, MM-GBSA binding free energy analysis, per-residue energy decomposition, *in silico* ADMET profiling, and complementary *in vitro* cellular and biophysical assays, supported by experimental findings, was used to evaluate NAR as a dual-target modulator of Aβ aggregation and RAGE-associated inflammatory signaling. Within this framework, NAR was predicted to interact with key residues in the Aβ42 groove, including PHE_20, PHE_19, and HIS_13, and to engage the RAGE interface through interactions involving ARG_98, LYS_52, and LYS_110. These computational observations were supported by experimental findings showing suppression of Aβ42 aggregation, reduced fibril seeding competence, attenuation of Aβ-induced NF-κB activation, and protection against Aβ-associated cellular toxicity.

Each analytical layer complemented the others: docking-defined binding modes were dynamically evaluated via MD simulations, MM-GBSA provided an energetic context for the observed interactions, and per-residue decomposition identified residues that contributed most strongly to ligand engagement. The ADMET analysis provided a comparative assessment of physicochemical and pharmacokinetic properties, although these predictions require experimental validation and should not be interpreted as direct indicators of *in vivo* efficacy or safety. In particular, ALZ-801 was included primarily as a clinically relevant comparative reference compound rather than as a novel candidate undergoing primary ADMET evaluation.

NAR demonstrated favorable predicted drug-likeness properties, including compliance with Lipinski criteria, predicted BBB accessibility, and comparatively balanced physicochemical characteristics. However, liabilities, including high plasma protein binding and predicted CYP2D6 substrate probability, remain important considerations for future optimization. Overall, the combined computational and experimental findings support the further investigation of NAR as a potential lead scaffold for multi-target therapeutic strategies targeting both amyloid aggregation and RAGE-associated neuroinflammatory signaling in AD.

## Data Availability

The original contributions presented in the study are included in the article/Supplementary Material. Additionally, the complete molecular dynamics simulation, MM-GBSA binding free energy calculations, and per-residue decomposition analysis for all 10 replicates are publicly available on Zenodo at https://doi.org/10.5281/zenodo.20527834. Further inquiries can be directed to the corresponding author.
