# Supplementary material for: Naringenin as a dual-target modulator of RAGE–NF-κB signaling and Aβ fibrillization in Alzheimer’s disease: integrated computational and experimental analyses
Source: Front Pharmacol. 2026 Aug 25;17:1901236. doi: 10.3389/fphar.2026.1901236 (PMC13550140; doi:10.3389/fphar.2026.1901236)
Supplement: Supplementary file 1 [file Supplementaryfile1.docx]

Supplementary Material

# 1 Supplementary Figures and Tables

## 1.1 Supplementary Tables

**Supplementary Table 1**. Summary of compound libraries screened against the RAGE structure (PDB ID: 6XQ3) during the virtual screening campaign. The table shows the total number of compounds screened from each library, the number of hits obtained after Standard Precision (SP) and Extra Precision (XP) docking, and the number of compounds with GlideScore values ≤ −5.0 kcal/mol selected for further analysis.

| Library | Library Size | Representative  Hit | SP Hits | XP Hits | GlideScore ≤ −5.0 kcal/mol |
| --- | --- | --- | --- | --- | --- |
| ChemDiv Drug Repurposing Library | 4541 | LNF-MP-993 | 280 | 28 | 23 |
| InterBio Natural Compounds Library | 485973 | LNF-MP-102 / NAR | 4383 | 483 | 426 |
| Enamine Fragment Library (Carboxylic Acid Fragments) | 4000 | HTS-Z-448 | – | 398 | 49 |
| InterBio Synthetic Compounds Library | 69040 | FN-EN-249 | 32880 | 3282 | 1034 |
| Enamine Targeted BACE Library | 7171 | HTS-Z-886 | 799 | 79 | 23 |
| Total | **570725** | – | **38342** | **4270** | **1559** |

**Supplementary Table 2**. Cytotoxicity evaluation of selected hit compounds identified from virtual screening against RAGE. Cytotoxic activity was assessed using the colorimetric MTS [3-(4,5-dimethylthiazol-2-yl)-5-(3-carboxymethoxyphenyl)-2H-tetrazolium] viability assay after 72 h compound exposure in human cancer cell lines (A549, CCRF-CEM, HCT116, HCT116 p53−/−, K562, and U2OS) and non-cancer cell lines (BJ and MRC-5). Measurements were performed using the CellTiter 96® AQueous One Solution reagent (Promega, USA) according to the manufacturer’s instructions and an established protocol (Agrawal, 2021). IC_50_ values are expressed in µM as mean ± SD (n ≥ 6). IC_50_ values > 50 µM were considered indicative of low or negligible cytotoxicity under the tested conditions, whereas IC50 values < 50 µM were considered indicative of weak to moderate cytotoxicity.

| Compound | A549 | CCRF-CEM | HCT11 | HCT116 p53-/- | K562 | U2OS | BJ | MRC-5 |
| --- | --- | --- | --- | --- | --- | --- | --- | --- |
| HTS-Z-886 | >50 | >50 | >50 | >50 | >50 | >50 | >50 | >50 |
| NAR | >50 | >50 | >50 | >50 | >50 | >50 | >50 | >50 |
| FN-EN-249 | >50 | 42.13 ± 4.34 | >50 | >50 | >50 | >50 | >50 | >50 |
| HTS-Z-448 | >50 | >50 | >50 | >50 | >50 | >50 | >50 | >50 |
| LNF-MP-102 | >50 | >50 | >50 | >50 | >50 | >50 | >50 | >50 |
| LNF-MP-993 | >50 | >50 | >50 | >50 | 43.31 ± 2.97 | >50 | >50 | >50 |

Agrawal, K. ed. (2021). *Laboratory Techniques in Cellular and Molecular Medicine*., 1st edition. Olomouc, Czech Republic: Palacký University Olomouc.

**Supplementary Table 3**. Molecular docking scores and key ligand–target interactions identified for RAGE (PDB ID: 6XQ3) and Aβ fibril (PDB ID: 2MXU) complexes. Docking scores are reported in kcal/mol. Interactions include hydrogen bonds, salt bridges, π-cation interactions, and π–π stacking interactions predicted from molecular docking analysis. V6Y and ALZ-801 were used as reference ligands for RAGE and Aβ fibril targets, respectively.

| Target Protein (PDB ID) | Ligand | Docking Score (kcal/mol) | Hydrogen Bond Interactions | Other Interactions |
| --- | --- | --- | --- | --- |
| RAGE (6XQ3) | V6Y | −7.033 | LYS_39, LYS_52, ARG_98, ASN_112 | Salt bridges: LYS_39, LYS_52; π-cation: LYS_110 |
| RAGE (6XQ3) | NAR | −5.072 | GLU_50, LYS_110 | π-cation: ARG_98, LYS_110 |
| Aβ fibril (2MXU) | ALZ-801 | −6.909 | HIS_A:14, HIS_B:14 | – |
| Aβ fibril (2MXU) | NAR | −8.098 | HIS_A:14 | π–π stacking: HIS_A:14 |

**Supplementary Table 4**. Protein-Ligand Interaction Profile analysis. The data summarizes the atomic-level recognition patterns, spatial distances (Å), and specific geometric attributes. Key interactions, including hydrophobic interactions, hydrogen bonds, π–π stacking, π-cation interactions, and salt bridges, were mapped for the RAGE (PDB ID: 6XQ3) and Aβ fibril (PDB ID: 2MXU) complexes. V6Y and ALZ-801 were used as reference ligands for RAGE and Aβ fibril targets, respectively.

| Protein-ligand complex | Interaction Type | Residue | Amino Acid | Distance (Å) | Details / Atoms involved |
| --- | --- | --- | --- | --- | --- |
| 6XQ3_V6Y | Hydrophobic | 21A | ALA | 3.62 | Ligand Atom: 3429; Protein Atom: 14 |
|  |  | 22A | MET | 3.92 | Ligand Atom: 3435; Protein Atom: 24 |
|  |  | 98A | ARG | 3.67 | Ligand Atom: 3425; Protein Atom: 1236 |
|  |  | 100A | GLN | 3.71 | Ligand Atom: 3419; Protein Atom: 1270 |
|  |  | 110A | LYS | 3.68 | Ligand Atom: 3425; Protein Atom: 1424 |
|  |  | 112A | ASN | 3.82 | Ligand Atom: 3437; Protein Atom: 1457 |
|  | Hydrogen | 112A | ASN | 3.26 (D-A) / 2.28 (H-A) | Angle: 161.97∘; Donor: Atom 1453 [Nam], Acceptor: Atom 3409 [O.co2] |
|  | π-cation | 110A | LYS | 3.65 | Offset: 1.54; Protein Charged: Yes; Aromatic Group (Atoms 3427–3432) |
|  | Salt bridges | 39A | LYS | 2.71 | Protein Positive: Yes; Carboxylate Group (Atoms 3408, 3409) |
|  |  | 52A | LYS | 3.37 | Protein Positive: Yes; Carboxylate Group (Atoms 3405, 3406) |
|  |  | 98A | ARG | 4.91 | Protein Positive: Yes; Carboxylate Group (Atoms 3405, 3406) |
| 6XQ3_NAR | Hydrophobic | 52A | LYS | 3.50 | Ligand Atom: 6663; Protein Atom: 516 |
|  |  | 98A | ARG | 3.97 | Ligand Atom: 6655; Protein Atom: 1203 |
|  |  | 98A | ARG | 3.69 | Ligand Atom: 6657; Protein Atom: 1205 |
|  |  | 98C | ARG | 3.92 | Ligand Atom: 6664; Protein Atom: 4476 |
|  |  | 99A | CYS | 3.93 | Ligand Atom: 6663; Protein Atom: 1225 |
|  |  | 100A | GLN | 3.67 | Ligand Atom: 6666; Protein Atom: 1239 |
|  |  | 100A | GLN | 3.31 | Ligand Atom: 6667; Protein Atom: 1237 |
|  |  | 110A | LYS | 3.88 | Ligand Atom: 6655; Protein Atom: 1392 |
|  | Hydrogen | 52A | LYS | 4.03 (D-A) / 3.31 (H-A) | Angle: 153.17∘; Donor: Atom 518 [N3+], Acceptor: Atom 6649 [O2] |
|  |  | 98A | ARG | 3.85 (D-A) / 3.09 (H-A) | Angle: 133.51∘; Donor: Atom 1208 [N3], Acceptor: Atom 6652 [O2] |
|  |  | 98C | ARG | 3.97 (D-A) / 3.30 (H-A) | Angle: 125.82∘; Donor: Atom 4477 [N2], Acceptor: Atom 6652 [O2] |
|  |  | 100A | GLN | 3.74 (D-A) / 3.24 (H-A) | Angle: 111.61∘; Donor: Atom 1240 [N3], Acceptor: Atom 6653 [O2] |
|  |  | 100C | GLN | 3.91 (D-A) / 3.11 (H-A) | Angle: 139.70∘; Donor: Atom 4509 [N3], Acceptor: Atom 6653 [O2] |
|  |  | 111A | SER | 3.62 (D-A) / 2.70 (H-A) | Angle: 150.38∘; Donor: Atom 1409 [N3], Acceptor: Atom 6650 [O2] |
|  |  | 111C | SER | 3.62 (D-A) / 3.14 (H-A) | Angle: 110.09∘; Donor: Atom 4478 [N3], Acceptor: Atom 6650 [O2] |
|  |  | 112A | ASN | 4.07 (D-A) / 3.48 (H-A) | Angle: 118.11∘; Donor: Atom 1420 [N3], Acceptor: Atom 6650 [O2] |
|  |  | 112A | ASN | 3.95 (D-A) / 3.46 (H-A) | Angle: 111.71∘; Donor: Atom 1427 [N3], Acceptor: Atom 6651 [O2] |
|  |  | 112C | ASN | 4.07 (D-A) / 3.27 (H-A) | Angle: 136.02∘; Donor: Atom 4489 [N3], Acceptor: Atom 6650 [O2] |
|  | π-cation | 52A | LYS | 4.13 | Offset: 1.87; Protein Charged: Yes; Aromatic Group (Atoms 6656, 6662, 6663, 6666–6668) |
|  |  | 52C | LYS | 4.22 | Offset: 1.70; Protein Charged: Yes; Aromatic Group (Atoms 6656, 6662, 6663, 6666–6668) |
|  |  | 98A | ARG | 3.59 | Offset: 0.43; Protein Charged: Yes; Aromatic Group (Atoms 6657, 6659, 6660, 6661, 6664, 6665) |
|  |  | 98C | ARG | 3.78 | Offset: 0.54; Protein Charged: Yes; Aromatic Group (Atoms 6657, 6659, 6660, 6661, 6664, 6665) |
|  |  | 110A | LYS | 4.78 | Offset: 1.88; Protein Charged: Yes; Aromatic Group (Atoms 6657, 6659, 6660, 6661, 6664, 6665) |
|  |  | 110C | LYS | 4.83 | Offset: 1.73; Protein Charged: Yes; Aromatic Group (Atoms 6657, 6659, 6660, 6661, 6664, 6665) |
| 2MXU_Alz-801 | Hydrogen | 14B | HIS | 2.85 (D-A) / 1.86 (H-A) | Angle: 166.19∘; Donor: Atom 5543 [Nam], Acceptor: Atom 537 [Nar] |
|  |  | 14B | HIS | 2.73 (D-A) / 1.71 (H-A) | Angle: 172.42∘; Donor: Atom 5574 [N3], Acceptor: Atom 537 [Nar] |
|  | Salt bridges | 14A | HIS | 4.58 | Protein Positive: Yes; Sulfonic acid (Atoms 5738, 5738, 5740, 5741, 5742) |
|  |  | 14B | HIS | 5.30 | Protein Positive: Yes; Sulfonic acid (Atoms 5738, 5738, 5740, 5741, 5742) |
| 2MXU_NAR | Hydrophobic | 12A | VAL | 3.36 | Ligand Atom: 5752; Protein Atom: 22 |
|  |  | 17A | LEU | 3.44 | Ligand Atom: 5756; Protein Atom: 112 |
|  |  | 17B | LEU | 3.69 | Ligand Atom: 5752; Protein Atom: 590 |
|  |  | 32A | ILE | 3.61 | Ligand Atom: 5755; Protein Atom: 329 |
|  |  | 32B | ILE | 3.45 | Ligand Atom: 5744; Protein Atom: 807 |
|  | Hydrogen | 14A | HIS | 3.67 (D-A) / 2.73 (H-A) | Angle: 170.46∘; Donor: Atom 5742 [O2], Acceptor: Atom 59 [Nar] |
|  |  | 33C | GLY | 3.73 (D-A) / 3.11 (H-A) | Angle: 124.69∘; Donor: Atom 5740 [O2], Acceptor: Atom 1302 [O2] |
|  | π–π stacking | 14A | HIS | 4.97 | Angle: 86.71∘; Offset: 0.69; Stacking Type: T; (Ligand Atoms 5745, 5751, 5752, 5755, 5756, 5757) |
|  | π-cation | 14B | HIS | 4.39 | Offset: 1.79; Protein Charged: Yes; Aromatic Group (Atoms 5746, 5748, 5749, 5750, 5753, 5754) |

## 1.2 Supplementary Figures

**Supplementary Figure 1.** **Initial screening of selected virtual screening hit compounds for inhibition of Aβ42-induced RAGE–NF-κB signaling.**

NF-κB luciferase reporter activity in RAGE-positive C6 glioma cells pre-treated with the indicated compounds (0.1–10 µM, 30 min) followed by stimulation with Aβ42 peptide for 6 h. Reporter activity was normalized to Aβ42-stimulated controls. BMS-345541 was included as a positive inhibitory control. Among the screened compounds, NAR showed the strongest concentration-dependent suppression of Aβ42-induced NF-κB activation, whereas the remaining compounds produced limited or negligible inhibitory effects under the tested conditions. Data are presented as mean ± SEM from four independent biological replicates.

**Supplementary Figure 2. Root mean square deviation (RMSD) analysis of protein backbone and ligand stability during 100 ns molecular dynamics simulations.**

Protein Cα RMSD (left panel) and ligand RMSD (right panel) profiles obtained from 100 ns molecular dynamics simulations of RAGE (PDB: 6XQ3) and Aβ fibril (PDB: 2MXU) complexes with reference ligands and NAR. Simulated complexes included RAGE–V6Y (black), RAGE–NAR (red), Aβ fibril–ALZ-801 (blue), and Aβ fibril–NAR (green). Protein RMSD plots monitor structural stability and conformational equilibration of the protein backbone throughout the simulation, whereas ligand RMSD plots reflect ligand mobility and stability within the binding interface. All complexes maintained overall structural stability during the simulation period, with moderate fluctuations consistent with dynamic ligand accommodation and conformational adaptation within the binding sites. Increased ligand RMSD observed for the Aβ fibril–ALZ-801 complex reflects higher ligand mobility relative to the other simulated systems.

**Supplementary Figure 3. Root mean square fluctuation (RMSF) analysis of residue flexibility during 100 ns molecular dynamics simulations.**

Protein Cα RMSF profiles of RAGE (PDB: 6XQ3) and Aβ fibril (PDB: 2MXU) complexes obtained from 100 ns molecular dynamics simulations with reference ligands and NAR. Simulated systems included RAGE–V6Y (black), RAGE–NAR (red), Aβ fibril–ALZ-801 (blue), and Aβ fibril–NAR (green). RMSF analysis was used to evaluate residue-wise flexibility and local conformational fluctuations throughout the simulation trajectories.

The RAGE complexes showed moderate fluctuations primarily within surface-exposed loop regions, while the overall backbone remained structurally stable. The Aβ fibril systems exhibited localized fluctuations mainly at terminal and turn-region residues, consistent with the intrinsically dynamic nature of amyloid fibril ends. Overall, the RMSF profiles indicate that ligand binding did not induce major destabilization of either the RAGE receptor or Aβ fibril structures during the simulation period.

**Supplementary Figure 4. Uncropped Western blot image corresponding to Fig. 3a.**

Uncropped Western blot images showing RAGE expression (left panel) and β-actin loading control (right panel) in C6 glioma cells and CHO cells. Lanes 1 and 2 correspond to the samples presented in Fig. 3a, while lane X represents an additional lane not included in the cropped main figure. A distinct ~38 kDa RAGE band is detected in C6 cell lysates but is absent in CHO cells. β-Actin was used as the loading control.

**Supplementary Figure 5. Structural snapshots comparing the relative orientation and local interaction networks of NAR and reference ligands within the target binding interfaces of RAGE and Aβ42.**

The RAGE receptor binding pocket (residues in pink) is shown in (a) V6Y-bound (cyan) and (b) NAR-bound (cyan) conformations, while the Aβ42 fibril structure (residues in blue) is shown in (c) ALZ-801-bound (orange) and (d) NAR-bound (orange) conformations. The dotted lines represent hydrogen bonds (green), H-π interactions (pink), and van der Waals (vdW) contacts/clashes (yellow). (b, d) Interaction mapping confirms that NAR samples a similar conformational space and target network as V6Y at the RAGE interface (engaging ARG_98 and LYS_52), while uniquely interacting with the aromatic sub-pocket (PHE_19, PHE_20) of Aβ42 compared to ALZ-801.

# 2 Supplementary Data: Parkinson’s Disease–Related Validation of RAGE Modulation

**2.1	Methods**

Differentiated SH-SY5Y dopaminergic-like cells were used to evaluate whether RAGE-associated modulation also exerts protective effects in a rotenone-induced Parkinson’s disease-related cellular stress model. SH-SY5Y cells were differentiated using retinoic acid and TPA treatment before compound exposure. Cells were pre-treated with RAGE-targeting compound NAR for 24 h, followed by rotenone exposure to induce dopaminergic toxicity and α-synuclein-associated stress. Cell viability was evaluated using the Alamar Blue assay. Gene expression analysis of tyrosine hydroxylase (TH), α-synuclein (SNCA), and RAGE was performed by RT-qPCR. Protein-level changes in TH, phosphorylated α-synuclein (pSer129), and RAGE were analyzed by immunocytochemistry using fluorescence microscopy. Statistical analysis was performed using one-way ANOVA followed by Tukey’s post hoc test.

**2.2	Results**

***2.2.1	RAGE-Targeting Compounds Protect Differentiated SH-SY5Y Cells from Rotenone-Induced Toxicity***

To investigate whether RAGE-associated modulation may also protect the Aβ context, selected compounds identified from the RAGE screening workflow were evaluated in differentiated SH-SY5Y dopaminergic-like cells exposed to rotenone. Initial toxicity screening demonstrated that NAR was well-tolerated across the tested concentration range **(Supplementary Figure 6A–C)**.

Pre-treatment with NAR significantly improved cell viability following rotenone exposure, particularly at 4–10 µM concentrations **(Supplementary Figure 6B-C)**. These findings indicate that the selected RAGE-targeting compound can attenuate dopaminergic stress in a Parkinson’s disease-related cellular model.

***2.2.2	NAR Modulates Dopaminergic and α-Synuclein-Associated Markers Following Rotenone Exposure***

Because NAR showed a consistent protective profile in the viability assay, it was selected for downstream molecular analysis. RT-qPCR demonstrated that rotenone exposure significantly reduced TH expression while increasing SNCA and RAGE transcript levels relative to untreated controls. Pre-treatment with NAR partially restored TH expression and reduced rotenone-associated increases in SNCA and RAGE expression.

***2.2.3	NAR Reduces Phosphorylated α-Synuclein and RAGE Expression in Rotenone-Treated Cells***

Immunocytochemical analysis further supported the protective effects of NAR in rotenone-treated SH-SY5Y cells. Rotenone exposure reduced TH immunoreactivity while increasing phosphorylated α-synuclein (pSer129) and RAGE staining intensity **(Supplementary Figure 7A–H)**. NAR pre-treatment restored TH expression and reduced both phosphorylated α-synuclein and RAGE immunoreactivity relative to rotenone-treated controls **(Supplementary Figure 7A–H)**. Together, these findings support the involvement of RAGE-associated signaling pathways in rotenone-induced dopaminergic stress and α-synuclein-associated cellular pathology.

**2.3	Discussion**

Although the primary focus of the present study was the dual modulation of Aβ aggregation and RAGE-mediated inflammatory signaling by NAR, RAGE signaling has also been implicated in α-synuclein-associated neurodegenerative dementia and dopaminergic dysfunction (Jiang et al., 2018; Long et al., 2022; Gasparotto et al., 2024). Therefore, supplementary experiments were performed in a rotenone-induced Parkinson’s disease-related cellular model to evaluate whether RAGE-associated modulation may extend beyond Aβ-driven pathology.

Rotenone exposure induced characteristic pathological changes in differentiated SH-SY5Y dopaminergic-like cells, including reduced TH expression, increased RAGE expression, and elevated phosphorylated α-synuclein levels, consistent with previous reports describing rotenone-mediated dopaminergic stress and α-synuclein pathology (Wang et al., 2020; Ramalingam et al., 2024). Among the tested compounds, NAR demonstrated the most consistent protective profile by attenuating rotenone-induced toxicity, restoring TH expression, and reducing phosphorylated α-synuclein and RAGE levels (Supplementary Figures S6 and S7).

These findings complement the Aβ-focused observations presented in the main manuscript by supporting the broader concept that RAGE functions as a convergent inflammatory signaling hub across multiple protein-misfolding disorders. While these experiments were not incorporated into the computational workflow of the present study, they provide additional biological evidence supporting the therapeutic relevance of targeting RAGE-associated pathways in neurodegenerative disease.

**2.4	References**

Gasparotto, J., Somensi, N., Girardi, C. S., Bittencourt, R. R., de Oliveira, L. M., Hoefel, L. P., et al. (2024). Is it all the RAGE? Defining the role of the receptor for advanced glycation end products in Parkinson’s disease. *J Neurochem* 168, 1608–1624. doi: 10.1111/jnc.15890

Jiang, X., Wang, X., Tuo, M., Ma, J., and Xie, A. (2018). RAGE and its emerging role in the pathogenesis of Parkinson’s disease. *Neurosci Lett* 672, 65–69. doi: 10.1016/j.neulet.2018.02.049

Long, H., Zhang, S., Zeng, S., Tong, Y., Liu, J., Liu, C., et al. (2022). Interaction of RAGE with α-synuclein fibrils mediates inflammatory response of microglia. *Cell Rep* 40, 111401. doi: 10.1016/j.celrep.2022.111401

Ramalingam, M., Jang, S., Kim, S., Bai, H., Jeong, G., Kim, B. C., et al. (2024). Rotenone and Its Derivative, Rotenoisin A, Induce Neurodegeneration Differentially in SH-SY5Y Cells. *Biomedicines* 12, 1703. doi: 10.3390/biomedicines12081703

Wang, X., Sun, X., Niu, M., Zhang, X., Wang, J., Zhou, C., et al. (2020). RAGE Silencing Ameliorates Neuroinflammation by Inhibition of p38-NF-κB Signaling Pathway in Mouse Model of Parkinson’s Disease. *Front. Neurosci.* 14, 353. doi: 10.3389/fnins.2020.00353

**Supplementary Figure 6. Experimental design and evaluation of NAR on SH-SY5Y cell viability in the presence and absence of rotenone.**

(A) Schematic representation of the experimental workflow. SH-SY5Y cells were differentiated into a dopaminergic-like phenotype, followed by treatment with NAR on day 8 for 24 hours. Subsequently, cells were exposed to rotenone (15 µM) for 24 hours. After treatments, cell viability, RT-PCR, and immunocytochemistry (ICC) analyses were performed. (B) Cell viability of SH-SY5Y cells treated with varying concentrations of NAR (0.1–100 µM) for 24 hours. (C) Effect of NAR pretreatment (0.01–20 µM) on rotenone (15 µM)-induced cytotoxicity.

**Supplementary Figure 7. Immunocytochemical and RT-PCR analysis of TH, phosphorylated α-synuclein, and RAGE protein expression following NAR pretreatment and rotenone exposure in differentiated SH-SY5Y cells.**

(A) Representative immunofluorescence images showing Tyrosine Hydroxylase (TH) expression. (B) Quantification of TH immunofluorescence. (C) Relative mRNA expression levels of Tyrosine Hydroxylase (TH) in NAR-pretreated cells. (D) Representative images showing co-immunostaining of phosphorylated α-synuclein (p-synuclein) and Receptor for Advanced Glycation End Products (RAGE). (E, G) Quantification of p-synuclein and RAGE immunofluorescence intensity, respectively. (F, H) Relative mRNA expression levels of SNCA and RAGE in NAR-pretreated cells, respectively. Data were reported as mean ± SEM (n=3 for RT-PCR; n=4 for ICC). *P < 0.05, **P < 0.01, ***P < 0.001 indicates statistical significance as compared to the rotenone treated SH-SY5Y cells. ###P < 0.001 indicates the statistical significance as compared to control SH-SY5Y cells. P < 0.05 was considered statistically significant.
